# Artificial intelligence based hybrid solar energy systems with smart materials and adaptive photovoltaics for sustainable power generation

**DOI:** 10.1038/s41598-025-01788-4

**Published:** 2025-05-19

**Authors:** Udit Mamodiya, Indra Kishor, Ramakrishna Garine, Priyam Ganguly, Nithesh Naik

**Affiliations:** 1 Faculty of Engineering and Technology, Poornima University, Sitapura, Jaipur, Rajasthan 303905 India; 2 Department of Computer Engineering, Poornima Institute of Engineering and Technology, Sitapura, Jaipur, Rajasthan 303905 India; 3 University of North Texas, Denton, TX 76205 USA; 4 Widener University, Chester, PA 19013 USA; 5 Department of Mechanical and Industrial Engineering, Manipal Institute of Technology, Manipal Academy of Higher Education, Manipal, Karnataka 576104 India

**Keywords:** Hybrid solar energy, AI-driven optimization, Smart materials, Adaptive photovoltaics, Blockchain smart grid, Reinforcement learning, Sustainable power generation, Energy science and technology, Engineering, Mathematics and computing

## Abstract

The advancement of solar energy systems requires intelligent, scalable solutions that adapt to dynamic environmental conditions. This research proposes a novel AI-enhanced hybrid solar energy framework integrating spatio-temporal forecasting, adaptive control, and decentralized energy trading. The core objective is to improve the efficiency, responsiveness, and scalability of solar power generation using a unified multi-layer architecture. The system comprises a CNN-LSTM model for accurate solar irradiance forecasting, reinforcement learning for real-time dual-axis tracking, and Edge AI for low-latency control decisions. To enhance optical and thermal efficiency, the design incorporates hybrid nanocoatings with self-cleaning and anti-reflective properties, along with dual-layer phase-change materials for real-time heat regulation. Additionally, adaptive perovskite-silicon photovoltaic cells were implemented to dynamically tune electrical characteristics such as bandgap and voltage based on irradiance levels. A blockchain-enabled smart grid facilitates secure and decentralized peer-to-peer energy transactions. Experimental validation was conducted over a full year at Sitapura, Jaipur (India), under real-world climatic conditions. The proposed system achieved a 41.4% increase in annual energy yield, an 18.7% improvement in spectral absorption efficiency, and an 11.9 °C reduction in average panel temperature compared to conventional MPPT and static PV setups. Furthermore, blockchain integration reduced energy dispatch latency from 180 to 48 ms, and AI-based hybrid storage management increased battery lifespan by over 60%. The framework demonstrates significant performance enhancement, real-time adaptability, and deployment viability, offering a transformative step toward intelligent, resilient, and sustainable solar energy systems.

**Supplementary Information:**

The online version contains supplementary material available at 10.1038/s41598-025-01788-4.

## Introduction

The growing global demand for sustainable and clean energy has propelled international research into solar photovoltaic (PV) systems with more advanced designs. Solar power continues to be a leading renewable energy source owing to its copious availability, scalability, and decreasing costs. Nevertheless, solar energy systems have several limitations in terms of their efficiency, dependability, and long-term sustainability. The major challenges are suboptimal energy conversion efficiency, fluctuating energy supply, thermal degradation, and inefficient energy storage and delivery. To address these issues, scientists are working on novel AI-based control systems, incorporating smart materials and adaptive photovoltaics to enhance the energy output and system robustness^1^. This study proposes a hybrid solar power system aided by AI that incorporates high-performance solar tracking, intelligent PV technologies, and blockchain-integrated smart grid integration for an efficient and scalable scheme for clean power production. Solar tracking is crucial for maximizing the usage of solar panels, because it allows them to be directly exposed to the sun. Solar panels placed on a fixed tilt cannot work at their optimal angles during the daytime, resulting in colossal energy loss^2^. To overcome this issue, several types of single-axis and dual-axis tracking systems exist that are dependent on pre-calculated algorithms that do not adapt to variable conditions, such as cloudiness, shadows, or atmospheric conditions. Traditional tracking systems face the problems of poor positioning, mechanical failure, and high energy consumption, rendering them ineffective under dynamic conditions. In response, researchers have explored deep learning and reinforcement learning methods for optimizing solar tracking and panel control, respectively^3,4^. This study proposes hybrid AI solar tracking based on CNNs, LSTMs, and RL to enhance the prediction and autonomous control of solar irradiance with dual-axis tracking. Unlike traditional methods, the system can learn by itself and adapt in real time; therefore, it can collect the maximum solar energy, even if the weather changes. Apart from tracking, another important parameter that determines photovoltaic (PV) efficiency is the interaction of light with photovoltaic materials. Conventional silicon-based PV cells are plagued by reflection loss, surface soiling, and overheating, which degrade their performance over time^5^. New materials, such as hybrid nano coatings and phase change materials (PCMs), have been considered for their potential use in enhancing light absorption and panel temperature control^6^. Quantum-dot-enhanced nano coatings can enhance the solar absorption efficiency by tailoring the spectral response of the solar cell, and anti-reflective coatings are used to minimize reflection-related losses. Moreover, PCMs ensure thermal stability by retaining excess heat, preventing panel overheating, and maximizing the long-term performance^7^. While the individual contributions of these materials have been viewed in previous studies, their synergistic impact on PV efficiency has not yet been studied. The main aim of the study is to combine hybrid nano coatings with dual-layer PCMs, thereby presenting a new, multi-functional solution for solar panel efficiency and reliability enhancement. In addition to material design and tracking schemes, adaptive photovoltaic systems have been used as game changers for maximizing solar energy conversion efficiency. The fixed electrical parameters of traditional PV modules restrict their adaptation to changes in solar irradiance, shading, and temperature^8^. Recent advancements in perovskite-silicon tandem solar cells have been proven to offer high efficiency and cost-effectiveness but are still defined by static performance under varying conditions^9,10^. This work presents an AI-optimized perovskite-silicon tandem PV system in which machine learning algorithms dynamically optimize the layer thickness, bandgap modulation, and electrical conductivity in real-time environmental conditions to achieve maximum power generation^11,12^. To further enhance energy efficiency, the current study suggests an AI-based real-time energy management system that switches dynamically between lithium-ion and supercapacitor storage based on real-time supply and demand power scenarios. The study proposed here has a number of innovative features that distinguish it from current solar energy optimization methods. First, in this research, a hybrid AI system that integrates CNN-LSTM-based solar irradiance prediction, RL-based dual-axis tracking, and Edge AI-based real-time decision-making is used^13,14^. In contrast to traditional tracking systems with predefined movement algorithms, the self-adaptive AI system dynamically adjusts to external changes to maximize the orientation of solar panels to capture energy. Second, the study proposes a multi-functional smart material improvement strategy involving hybrid nano coatings with self-cleaning, anti-reflective, and quantum-dot-based characteristics, and dual-layer phase change materials (PCMs) for enhanced thermal management^15^. Although nano coatings and PCMs have been studied individually, their combined effects on photovoltaic efficiency are unknown. This study creates a new synergy between material science and photovoltaic optimization, resulting in enhanced energy conversion rates and longer panel lives. A second major novelty is the creation of an adaptive photovoltaic system, where perovskite-silicon hybrid solar cells are dynamically optimized using real-time AI algorithms. Compared to conventional PV cells, which function under static conditions, the proposed system dynamically alters its electrical and optical characteristics in accordance with varying solar irradiance and temperature^16^. This auto-tuning enables the system to deliver greater energy output and enhanced efficiency over varied climatic scenarios. Moreover, this study proposes an industry-academy-collaborated AI integrated blockchain-based smart grid for secure and decentralized energy trading^17^. In contrast to conventional centralized grid systems, smart contracts were employed in the proposed framework to automate energy trading, and AI-enabled predictive switching was used to switch between lithium-ion batteries and supercapacitors for effective energy storage utilization^18^. This approach is novel and scalable, as it has not been studied before with this combination, including blockchain security, AI energy optimization, and hybrid energy storage in their own columns under one framework for internodal energy transaction under VE BIT AG smart grid integration^19^. This study constructed a holistic, intelligent, and high-efficiency hybrid solar energy system based on AI-driven solar tracking, smart material-based PV enhancement, adaptive photovoltaics, and blockchain-secured energy management, which is scalable and sustainable.

## Literature review

The unveiling of solar PV systems is due to the imperatives of heightened efficiency and reliability, along with the ability to respond better to dynamic environmental conditions. Mechanical devices are used in conventional solar trackers that facilitate the tilting of solar panels for maximum light exposure^20^. A single-axis tracker is able to tilt the panel horizontally in an east–west or north–south manner, while a two-axis tracker enables tilting on both sides for maximum exposure to sunlight during the day. They are known to provide larger energy output than fixed photovoltaic (PV) panels, however, they have a certain limitation in terms of the flexibility of responding to ever-changing weather conditions, causing the overall efficiency to be low^21^. Solar tracking systems have recently become more prominent in estimating the location of the sun through astronomical calculations on an adopt-to-rules basis^22,23^. In optimizing solar tracking systems, Artificial Neural Networks (ANNs) have been widely used for forecasting solar irradiance. As for the first application of an ANN model, the challenge arose in terms of overfitting, high difficulty level, and weak generalization of variable environmental conditions^24^. More recently, applications centered on prediction models, such as future convolutional neural networks, CNN, long short-term memory, and LSTM networks, have emerged as promising candidates for solar forecasting and panel movement prediction. CNN models learn spatial features from satellite imagery and irradiance data, whereas LSTM networks learn the temporal relationships associated with weather variations^25^. The combination of these approaches has led to an increased accuracy in solar irradiance forecasting^26,27^. Nonetheless, current CNN-LSTM solar tracking models are usually integrated independently of physical tracking systems, resulting in limited end-to-end integration between forecasting and panel movement optimization. Reinforcement Learning (RL)-based tracking models have also become popular because they allow solar panels to optimize their orientation independently over time^28^. RL methods employ trial-and-error learning to progressively improve panel positioning for an optimal energy output. However, there are no hybrid AI-integrated RL-based tracking systems that operate under uncertain environmental conditions^29^. This study introduces a hybrid AI system that combines CNN-LSTM prediction with RL-based tracking and Edge AI for real-time decision-making, building an intelligent, self-adaptive solar tracking system that can dynamically optimize solar panel orientation^30^. Over time, dust deposition and water droplets decrease light absorption, resulting in reduced power generation efficiency^31,32^. In response, researchers have examined smart nano coatings and antireflective coatings aimed at enhancing light absorption and self-cleaning. Quantum-dot-improved coatings have also been considered as a means of broadening the spectral range of PV cells to absorb near-ultraviolet and infrared light at useful wavelengths^33,34^. Such coatings enhance photon absorption and excitation of electrons, resulting in improved power conversion efficiency (PCE)^35^. Empirical studies suggest that for every 1 °C increase in temperature, the silicon-based PV panel efficiency decreases by approximately 0.5%^36^. While working on the solution to this problem, scientists have been exploring the possibilities of using Phase Change Materials (PCMs) as thermal managing materials^37,38^. Single-layer PCM systems can potentially stock surplus heat during high-sunlight hours and expel it during low-sunlight hours, maintaining a constant panel’s temperature constant^39^. However, single-layer PCMs have a limited thermal storage capacity and are prone to repeated energy discharge cycles. More recent research has focused on dual-layer and multilayer PCM systems to attain improved thermal storage capability^40^. In this setup, low-melting-point PCMs initially absorb heat, whereas higher-melting-point PCMs accumulate surplus energy to yield improved thermal regulation times^41^. Although such technology is promising, the combined effect of hybrid nano coatings and dual-layer PCMs on PV system efficiency remains to be investigated in detail^42,43^. This study recommends combining both technologies to produce a multifunctional smart-material-based enhancement system to sustain enhanced light absorption, thermal regulation, and surface hardness. Despite these advancements, current blockchain-based smart grids do not utilize the AI-powered predictive analytics necessary for real-time energy distribution and load balancing. Moreover, hybrid models of energy storage systems integrating lithium-ion batteries with supercapacitors have been suggested, yet without intelligent switch mechanisms that actively optimize energy utilization dynamically^44,45^. Metaheuristic algorithms such as Genetic Algorithms (GA), Particle Swarm Optimization (PSO), Grey Wolf Optimizer (GWO), and Ant Colony Optimization (ACO) have been extensively used in solar tracking and energy management^46,47^. These methods allow for smart decision making by continually optimizing system performance in accordance with real-time environmental factors. Recent advancements in deep-learning-driven hybrid optimization models have shown significant potential for improving solar energy efficiency^48^. Hybrid systems that combine deep learning (DL) and heuristic optimization approaches facilitate adaptive control systems, such that solar tracking, energy regulation, and storage optimization are dynamic through continuous real-time feedback from the environment. For instance, CNN-LSTM systems have been combined with PSO trackers, which allow solar panels to forecast the sun intensity and rotate dynamically^28^. Similarly, RL-based controllers have been tuned with GA-based fitness functions to ensure that tracking decisions are progressively improved for optimal energy output. Nevertheless, these models remain confined to theoretical analysis, with limited experimental validation in real-world applications reported in the literature. Faults in PV panels, inverters, and energy storage units can significantly undermine the efficiency of a system, causing unplanned power losses and shortening the lifespan of the system^21,49^. Ongoing research on AI-based predictive maintenance models-a systematic overview used for solar power systems-is typically based on machine learning and deep learning algorithms to help identify anomalies. Supervised learning models, including SVMs, RF, and Gradient Boosting Algorithms, have been used for diagnosing and classifying PV faults^37^. These techniques use past operating data, temperature fluctuations, and voltage changes to forecast failures prior to their occurrence. However, traditional machine learning models require large, labelled datasets to train on; therefore, they are not suitable in the presence of rare or unknown faults^50,51^. Deep learning methods, particularly Convolutional Neural Networks (CNNs) and Recurrent Neural Networks (RNNs), have shown better fault detection accuracy by learning intricate features from PV performance data. Researchers have applied CNN-based image processing to identify hotspots and microcracks in PV modules through infrared thermography, allowing the early diagnosis of degradation^45,46^. Furthermore, Long Short-Term Memory (LSTM) networks have been employed to detect anomalies in real time in PV power output, locating unusual patterns that predict system failures. Despite these developments, current AI-based fault detection models tend to run in isolation from the overall energy management system, resulting in disconnected diagnostic structures that fail to include automatic corrective measures^38,51^. Additionally, the actual deployment of AI-based predictive maintenance is not extensive because most research is based on theoretical simulations instead of experimental validation. The study found that there is a lack of an Integrated AI-Driven Solar Tracking and Optimization Framework consolidates the research gaps identified from the literature and to clearly align them with our contributions. Table 1 presents a structured overview highlighting key limitations in existing work and how the proposed system addresses them.

**Table 1:** Summary of domain-specific research gaps identified in AI-based hybrid solar energy systems and corresponding proposed contributions.

Objective	Research gap	Evidence from literature	Proposed contribution
AI-driven solar tracking	Limited integration of predictive AI (CNN-LSTM, RL) for real-time solar tracking optimization	Existing models rely on static MPPT or sensor-based tracking; predictive adaptive control remains underexplored	Integrate CNN-LSTM forecasting with RL-based dual-axis tracking for accurate, adaptive solar optimization
Smart materials	Insufficient exploration of nanocoatings and PCMs for temperature and spectral absorption control	Current materials studies focus on individual effects; few explore synergistic effects of PCMs + nanocoatings	Employ hybrid nanocoatings (quantum-dot, self-cleaning) and PCMs to enhance efficiency and thermal stability
Adaptive photovoltaics	Lack of real-time responsive PV materials that self-adjust to dynamic solar conditions	Most PV advancements rely on static materials with fixed optical properties, lacking self-adjustment capabilities	Design AI-tuned perovskite-silicon PV cells that dynamically adjust based on irradiance and temperature
Blockchain integration	Minimal application of blockchain for decentralized energy management in hybrid PV systems	Blockchain use in energy systems is often theoretical or limited to smart meter simulations, not real PV-grid integration	Implement blockchain-enabled smart grid for secure, decentralized energy trading and hybrid storage management
Real-world testing and scalability	Absence of long-duration field validation studies under diverse climate and operational scenarios	Most studies are simulation-based or limited to lab-scale; no full-year dual-axis validation under real environmental loads	Conduct 12-month experimental validation using a real system deployed at Sitapura (Jaipur), ensuring practical scalability and reliability of the hybrid system

The table outlines technical limitations in solar tracking, smart materials, adaptive photovoltaics, blockchain integration, and real-world testing, along with the novel aspects addressed by the current work.

## Methodology

This study provides a paradigm for an artificial intelligence-driven hybrid solar power system, including optimized solar tracking with advanced technology, advanced photovoltaic (PV) systems initiated by smart materials, adaptive photovoltaic technologies, and blockchain-based smart grid systems. The methodology includes a quantitative or scientific aspect in terms of applying machine learning algorithms, running empirical experiments, and real-time implementation to increase the efficiency of solar energy, optimal storage of energy, and decentralized energy management. Data collection conducted over a period of 12 months (January 2024–January 2025) to appropriately address seasonal fluctuations for model training for AI and performance evaluation. Experimental activities will be carried out within a period of six months, such as PV material performance tests, AI-based tracking verification, and blockchain-based energy trading experiments.

In contrast to conventional approaches such as LSTM-based prediction, MPPT-based solar tracking, and grid coordination centrally, the novel model offers a multi-layer AI optimization strategy with significantly enhanced performance under real-world variability. CNN-LSTM was preferred over naive LSTM or ARIMA techniques because it can capture the spatial–temporal features to enhance the precision of solar irradiance forecasting. Traditional MPPT methods are non-adaptive to abrupt environmental change, whereas reinforcement learning learns the optimal tracking policy online adaptively. Moreover, decentralized smart grid with blockchain-based control replaces centralized grid control, allowing secure, transparent, and low-latency energy trading. The integration of adaptive PV technology with hybrid storage controlled by AI enables self-tuning on both generation and storage sides, resulting in greater reliability and scalability than fixed systems.

### Over view of proposed work

The AI-hybrid solar energy system presented here optimizes solar energy conversion, storage, and grid integration by integrating CNN-LSTM forecasting, reinforcement learning dual-axis tracking, and Edge AI real-time control. Unlike conventional systems, it can automatically adapt to climatic variations to optimize irradiance capture. Smart material integration, such as hybrid nanocoatings and dual-layer PCMs, enhances light absorption and thermal management. Dynamic reconfigurable perovskite-silicon PV cells dynamically reconfigure real time for maximum efficiency. AI-optimized hybrid energy storage (Li-ion + super capacitor) and blockchain-based decentralized energy trading maximize energy transfer. The end-to-end system is experimentally proven, with confirmation of scalability and field-deployment readiness.

### System architecture and workflow

The proposed hybrid solar energy system uses AI blends machine-learning-driven solar tracking, material upgrade with intelligence, adaptive photovoltaics, and energy management using blockchain into a common and intelligent platform for energy optimization.

#### System architecture

**Fig. 1:**
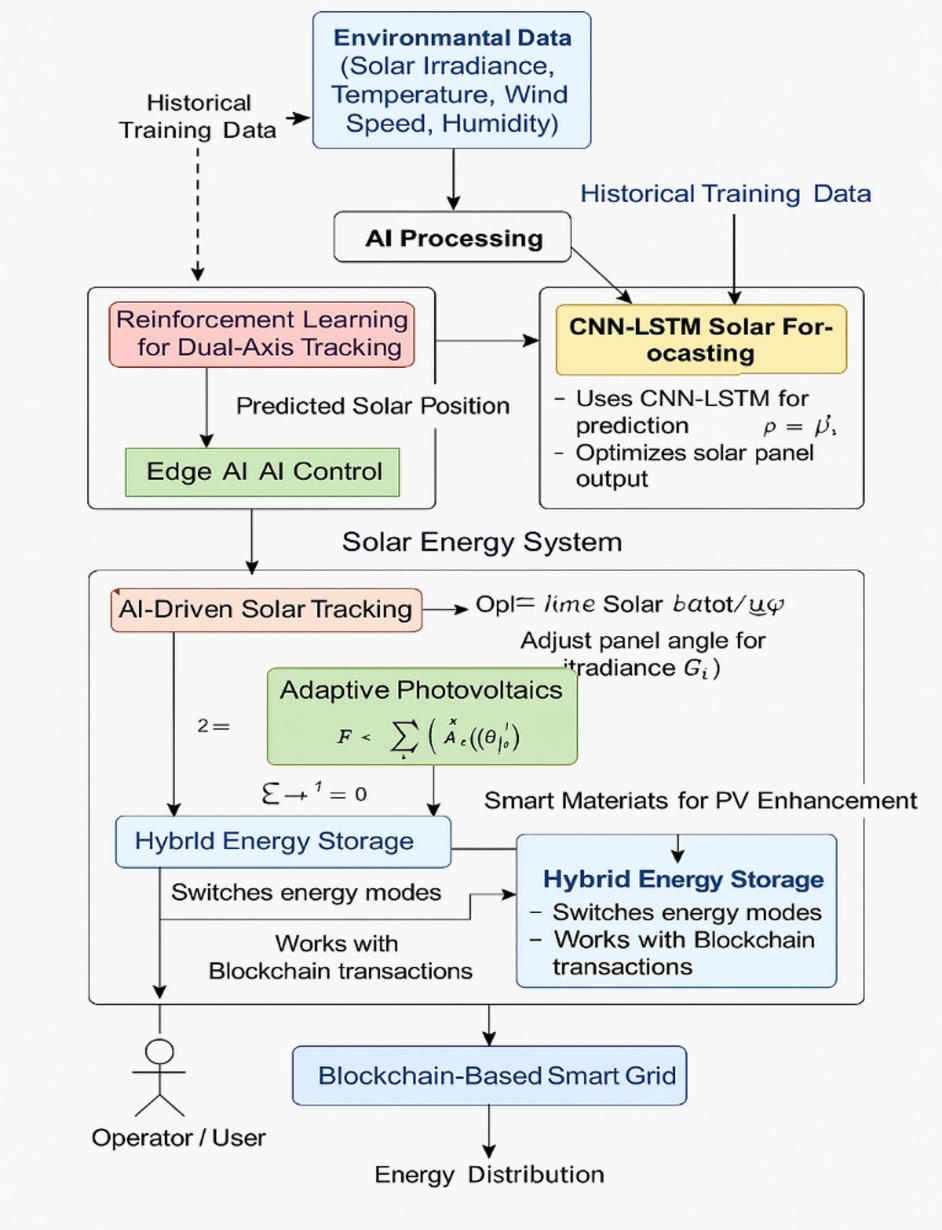
System architecture of AI-driven hybrid solar energy system.

The AI-based hybrid solar energy system integrates multiple integrated modules to enhance the decentralized energy management, energy conversion, and solar tracking. The system integrates CNN-LSTM solar irradiance forecasting, RL-based dual-axis tracking, and Edge AI for real-time applications to facilitate adaptive and efficient solar tracking. For PV efficiency improvement, smart materials such as hybrid nano coatings (self-cleaning, anti-reflective) and dual-layer Phase Change Materials (PCMs) are used to control the temperature and enhance light absorption. Additionally, adaptive perovskite-silicon hybrid PV cells dynamically adjust their electrical and optical properties to maximize the real-time energy yield. The system also includes an AI-driven hybrid energy storage system consisting of Li-ion batteries and supercapacitors, optimized through predictive charge–discharge balancing. The integration of blockchain-based smart grid technology enables secure decentralized energy trading, where AI-powered smart contracts facilitate real-time peer-to-peer (P2P) transactions. The system ensures self-learning and continuous optimization, thus improving overall efficiency and sustainability. Figure 1 shows the System Architecture of AI-Driven Hybrid Solar Energy System, which illustrates the integrated AI-driven hybrid solar energy framework combining solar tracking optimization, adaptive photovoltaics, smart materials, hybrid energy storage, and blockchain-based decentralized energy trading. The architecture begins with real-time environmental data collection, feeding into an AI-powered solar tracking system that utilizes CNN-LSTM for irradiance forecasting, reinforcement learning for dual-axis tracking, and Edge AI for real-time control execution. To enhance energy absorption and stability, the system incorporates smart materials, including hybrid nano coatings (self-cleaning, anti-reflective, and quantum-dot-based) and dual-layer Phase Change Materials (PCMs) for thermal regulation. adaptability and sustainability.

#### Workflow of the AI-driven hybrid solar energy system

The workflow follows a structured sequence for data acquisition, AI-based decision-making, and energy management. Initially, environmental parameters (solar irradiance, temperature, wind speed, and humidity) were collected from sensor networks and historical datasets. The CNN-LSTM model forecasts solar intensity, guiding the reinforcement learning-based dual-axis tracking system to dynamically adjust the panel angles. Edge AI processing ensures real-time execution with low latency and optimizes energy capture. Following solar tracking, smart materials enhance energy conversion efficiency by reducing reflection losses and stabilizing the temperature. The adaptive PV system self-tunes its properties by adjusting the bandgap and charge-transport mechanisms for the maximum energy output. Energy management is handled by an AI-integrated hybrid storage system, where predictive analytics optimize energy storage and discharge cycles. If the energy demand is high, AI prioritizes the direct grid distribution, whereas excess energy is securely traded via blockchain-enabled smart contracts. The system continuously monitors efficiency metrics, allowing for self-optimization in response to environmental fluctuations and market conditions.

**Fig. 2:**
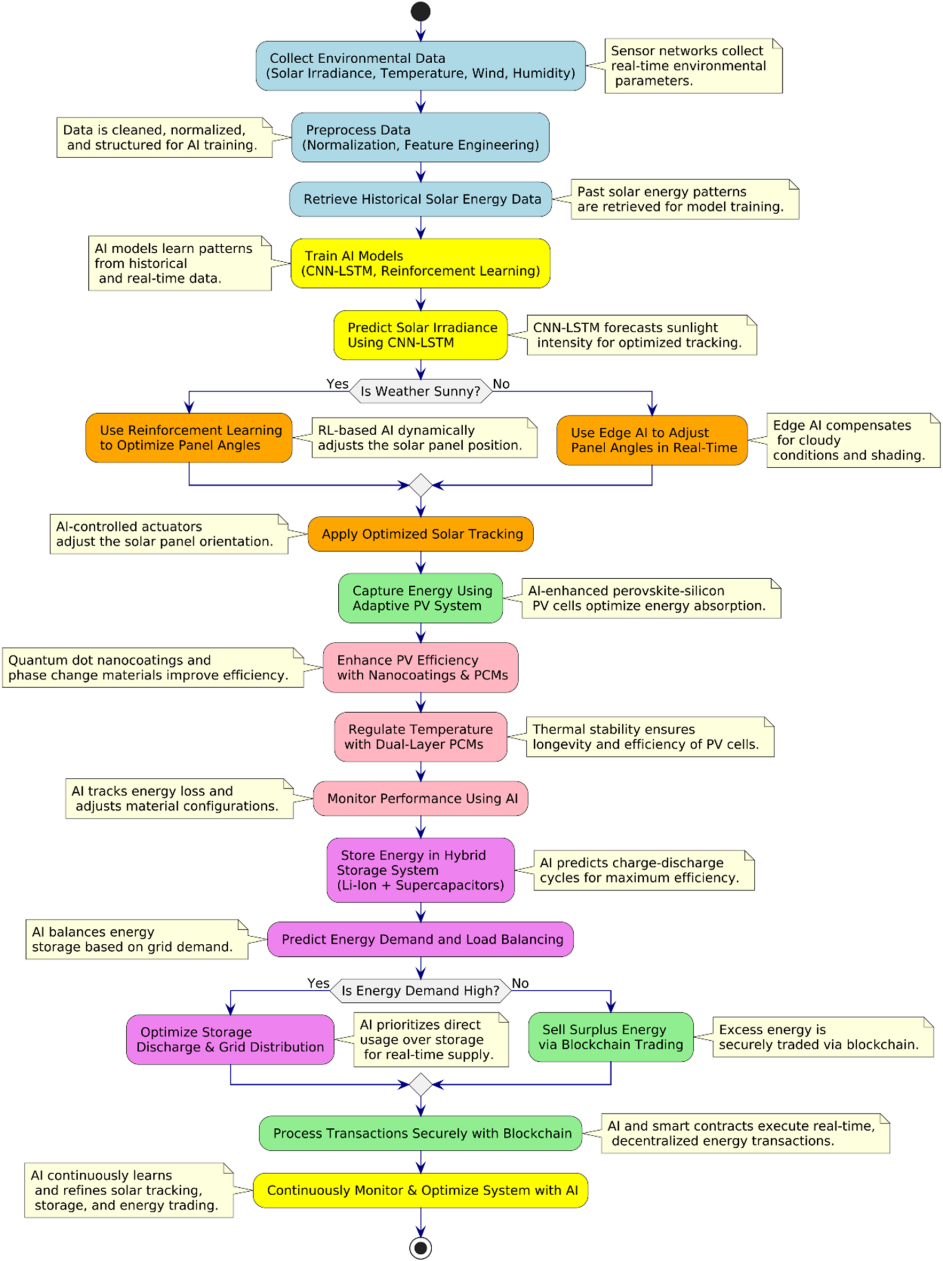
Workflow of the AI-driven hybrid solar energy system.

The workflow diagram in Fig. 2 outlines the sequential process of data acquisition, AI-based decision making, and energy management for real-time optimization and sustainability. The process begins with environmental data collection, in which sensors record solar irradiance, temperature, humidity, and wind speed, supplemented by historical datasets for AI model training. The CNN-LSTM model predicts solar intensity trends, enabling reinforcement learning (RL)-based dual-axis tracking to dynamically adjust the panel angles for maximum solar exposure. Edge AI ensures a low-latency execution, allowing instantaneous tracking corrections based on real-time environmental conditions. Following solar tracking, smart materials enhance energy absorption and stability by integrating hybrid nano coatings (anti-reflective, self-cleaning) and dual-layer Phase Change Materials (PCMs) for thermal regulation. Simultaneously, the adaptive photovoltaic system self-tunes its electrical and optimizing energy conversion under diverse environmental conditions. The AI-integrated hybrid energy storage system utilizes predictive analytics to manage charge–discharge cycles across Li-ion batteries and supercapacitors, ensuring efficient energy retention and distribution.

If the energy demand is high, AI prioritizes grid supply, whereas excess energy is securely traded using blockchain-enabled smart contracts, enabling decentralized, real-time energy transactions. Continuous AI monitoring and optimization refined tracking, storage, and trading processes, making the system intelligent, self-sustaining, and energy efficient.

### Dataset collection and preprocessing

A hybrid solar energy system powered by AI requires a high-quality, diverse, and well-organized dataset for training machine learning models, solar tracking optimization, and decentralized energy management strategy validation. A 12-month data acquisition period (January 2024–January 2025) was selected to record seasonal changes, real-time environmental factors, and past energy trends at the experimental site in Sitapura, Jaipur, Rajasthan, India (302022). Table S1 shows the Environmental Parameters and Energy Output Comparison for AI-Based and Conventional Solar Systems Dataset. This site is suitable for assessing solar energy efficiency under various climatic conditions, such as hot summers, monsoons, and winters. These data are utilized for CNN-LSTM-based solar irradiance prediction, reinforcement-learning-based solar tracking optimization, Edge AI-based real-time decision-making, and blockchain-based energy trading mechanisms. The gathered data is preprocessed, feature engineered, and thoroughly quality-improved prior to integration into AI-based models.

#### Data collection

A blend of real-time sensor networks, databases of past times, PV power monitoring systems, and blockchain-based energy transaction histories was utilized to construct a complete dataset. These data are critical for predictive modeling, real-time control, and benchmarking of the performance of an AI-integrated solar energy system.

##### Real-time environmental data

A real-time environmental dataset was collected for this study. The data collected from the on-site IoT-based sensor networks were installed at the experimental location. The collected data provides a critical parameter that influences solar energy generation and tracking efficiency.

- The solar irradiance (measured in W/m^2^) determines the amount of incident solar energy available for photovoltaic conversion.
- Ambient temperature (measured in °C)—Effects of solar panel efficiency and thermal regulation mechanisms.
- Relative humidity (measured in %)—The impacts of cooling efficiency and potential degradation of PV materials.
- Wind speed (measured in m/s) affects the panel stability and tracking optimization.
- The relationship between solar irradiance and PV power output is modeled as from Eq. (1):
  1 $${\text{P}}_{{{\text{PV}}}} = \eta {\text{PV}} \times {\text{A}}_{{{\text{PV}}}} \times {\text{I}}_{{{\text{solar}}}} \times (1 - \gamma \left( {{\text{T}}_{{{\text{ambient}}}} - {\text{T}}_{{{\text{ref}}}} } \right)$$

where PPV is the PV power output (W); ηPV is the efficiency of the PV system (%); APV is the effective surface area of the PV panel (m^2^); I_solar_ is the incident solar irradiance (W/m^2^); γ is the temperature coefficient of efficiency (%/°C); T_ambient_ is the real-time ambient temperature (°C); and T_ref_ is the standard reference temperature (°C).

##### Historical solar energy data

Historical solar energy data were retrieved from weather databases and solar radiation-monitoring stations. These data are crucial for training CNN-LSTM forecasting models because they provide long-term trends in solar radiation, seasonal variations, and cloud cover indices. The historical daily solar irradiance records enable long-term predictive analytics and Weather pattern analysis assists in reinforcement learning-based tracking optimization.

##### PV power output data

Photovoltaic power output data were collected from real-time monitoring of experimental PV panels deployed in Sitapura, Jaipur. These data are used to benchmark the efficiency of the AI-optimized solar tracking techniques compared to conventional tracking methods and Adaptive perovskite-silicon hybrid PV cell performance under varying environmental conditions.

##### Blockchain-based energy transaction logs

To validate the decentralized energy trading mechanisms, blockchain-based smart grid transaction logs were analyzed. The dataset includes Peer-to-peer (P2P) transactions of surplus solar energy within a smart grid network and AI-powered energy pricing models optimized for demand-response efficiency. This data set enables AI-based decision making for secure, real-time, decentralized energy distribution.

#### Data preprocessing

To enhance data quality and reliability, preprocessing techniques are applied to ensure smooth AI model training, noise reduction, and structured feature engineering.

##### Normalization and noise reduction

Real-world datasets contain sensor inconsistencies and fluctuations that must be corrected.

Min-max scaling is applied to bring all features to a normalized range between 0 and 1, as shown in Eq. (2):

2 $${\text{X}}^{\prime } = \frac{{{\text{X}} - {\text{X}}_{{{\text{min}}}} }}{{{\text{X}}_{{{\text{max}}}} - {\text{X}}_{{{\text{min}}}} }}$$

Gaussian filtering and statistical smoothing techniques are used to remove noise and inconsistencies.

##### Handling missing data

Missing values in real-time sensor data can affect machine learning model performance.

- Linear interpolation is used for short-term missing values.
- K-nearest neighbor (KNN) imputation is applied to time-dependent gaps in solar energy datasets.

##### Feature engineering for AI optimization

Feature engineering improves AI model efficiency by extracting key predictive attributes:

- Solar zenith angle (θ*z*)—Determines the sun’s position for tracking optimization.
- Air mass factor—Helps estimate atmospheric interference on solar radiation.
- Cloud cover index—Enhances CNN-LSTM forecasting accuracy.

#### Collected data summary

The structured dataset is summarized in Table 2, which provides an overview of the collected dataset including real-time environmental parameters, historical solar energy data, PV power output measurements, and blockchain-based energy transaction logs. These data sources are essential for CNN-LSTM-based solar irradiance forecasting, reinforcement-learning-based dual-axis tracking optimization, and AI-driven decentralized energy management. The structure of the dataset was designed such that accurate AI prediction can be achieved while efficiently using solar energy and optimizing the operations of the smart grid.

**Table 2:** Summary of collected data for AI-driven hybrid solar energy system.

Data type	Source	Frequency	Usage in AI framework
Solar irradiance (I_solar_, W/m^2^)	Real-time sensor data	1 min interval	CNN-LSTM forecasting for AI-based tracking
Temperature (T_ambient_, °C)	Real-time sensor data	1 min interval	Affects PV efficiency and adaptive material response
Humidity (H_rel_, %)	Real-time sensor data	1 min interval	Influences PV performance and cooling efficiency
Wind speed (V_wind_, m/s)	Real-time sensor data	5 min interval	Affects stability of tracking system
Power output from PV panels (P_PV_, kW)	PV system sensors	5 min interval	Benchmarking adaptive PV system
Historical solar energy data (X_hist_)	Weather databases	Daily	AI model training for long-term forecasting
Blockchain energy transaction logs (X_block_)	Smart grid network	Event-based	AI-optimized decentralized energy trading

The preprocessed data on which this study is based will serve as an entry point for AI model integration. The CNN-LSTM forecasting engine models the irradiance of solar radiation on structured data as its basis for forecasting, whereas RL learns to evolve a strategy for optimal dual-axis tracking in real time from the feedback loop that comprises the environmental factors. As AI plays a vital role in creating efficient grid management systems, energy transaction logs on the blockchain provide a complement. The next section describes the optimization of solar tracking following the AI approach, presenting the dataset whose optimization computation by AI occurs in a real-time scenario guaranteeing an optimized solar energy output.

### AI-driven solar tracking optimization

The accuracy of solar tracking systems plays an important role in their efficiency of solar energy systems. The proposed AI-based solar tracking system is a combination of CNN-LSTM-based irradiance prediction, RL-based dual-axis tracking, and Edge AI for the real-time implementation of this continuous and adaptive optimization. Some of the conventional techniques such as basic LSTM, ARIMA, fuzzy logic controllers, and static MPPT techniques were initially on the waiting list for solar irradiance tracking and forecasting. Such techniques were either devoid of spatial feature extraction (in the case of LSTM/ARIMA) or not environmentally adaptive in response (such as in the case of MPPT and fuzzy logic). The selected CNN-LSTM for spatial–temporal learning, Reinforcement Learning for environment-conscious dual-axis tracking optimization, and Edge AI for low-latency control. The trio in three ensures real-time, adaptive, and efficient performance that is optimal for smart solar energy systems to operate under dynamic climatic conditions.

#### Solar irradiance forecasting model (CNN-LSTM)

Solar irradiance forecasting is crucial for the pre-optimization of panel orientation. A hybrid deep learning model consisting of Convolutional Neural Networks (CNN) and Long Short-Term Memory (LSTM) networks was employed to predict future irradiance patterns, thereby enabling proactive solar tracking control. Forecasting involves feature extraction through a CNN, followed by time-series pattern analysis using LSTM. The model learns to reduce the difference between the predicted and actual solar irradiance values by utilizing the loss function, as given in Eq. (3).

3 $${\text{L}} = \frac{1}{N}{ }\mathop \sum \limits_{i + 1}^{N} { }\left( {{\text{I}}_{{{\text{solaractual}}}} \left( {\text{t}} \right) - {\text{I}}_{{{\text{solarpredicted}}}} \left( {\text{t}} \right)} \right)^{2}$$

where I_solaractual_ (t) = Measured solar irradiance at time ${\overline{\text{t}}}$, I_solarpredicted_ (t) = CNN-LSTM model output for solar irradiance, ${\overline{\text{N}}}$ = Total number of training samples.

The CNN layer extracts spatial features from historical weather and irradiance maps, whereas the LSTM layer models time-dependent variations, thereby enhancing the forecast accuracy.

#### Reinforcement learning for dual-axis solar tracking

A reinforcement learning-based approach was used to dynamically optimize the panel orientation. The tracking system learns to adjust azimuthal (θ_*az*_) and elevation (θ_el_) angles in real-time by continuously evaluating the solar irradiance received at different panel positions.

The tracking system is formulated as a Markov Decision Process (MDP), where:

- State (${\overline{\text{S}}}$_t_): Current panel position ($\overline{\theta }$_az_, $\overline{\theta }$_el_) and environmental conditions.
- Action (${\overline{\text{A}}}$_t_): Change in panel angles (Δ$\overline{\theta }$_az_, Δ$\overline{\theta }$_el_) based on AI decisions.
- Reward function (Rt): Energy gain from the panel’s new position.

The reward function is designed to maximize received solar energy shown in Eq. (4):

4 $${\text{Rt}} = {\text{I}}_{{{\text{solar}}}} \left( {\text{t}} \right) \times {\text{cos}}\left( {{\uptheta }_{{{\text{inc}}}} } \right)$$

where θ_*inc*_ is the incidence angle between the sun’s rays and the panel surface. The RL agent continuously adjusts tracking angles to maximize long-term energy yield.

#### Edge AI for real-time decision making

To eliminate tracking delays and reduce computational overhead, Edge AI technology is deployed. Unlike cloud-based models, Edge AI processes data locally, enabling real-time tracking adjustments without latency issues.

##### Implementation of edge AI in solar tracking

- Low-power AI models were deployed on embedded hardware for on-site tracking control.
- Lightweight neural networks process CNN-LSTM forecasts and RL tracking decisions within milliseconds.
- The proposed AI models dynamically switch between cloudy and sunny conditions, modifying the tracking response times based on edge-computed predictions.

#### Evaluation metrics for solar tracking performance

To quantify the performance of the AI-based solar tracking system, a set of performance metrics was utilized, enabling measurable improvements over traditional tracking systems. The (RMSE) is the performance metric used to quantify the accuracy of the CNN-LSTM model for forecasting solar irradiation, allowing a small divergence in values between the predicted or forecasted solar arrays and solar irradiance represented by the actual solar irradiance values. The methodologies to harvest energy gains by AI-based systems as ηgain effectiveness as a performance metric set forth the advantages of real-time reinforcement learning-based solar panel trackers over conventional fixed tilt solar panels. In other words, they serve as performance metrics that provide scientific evidence to justify the validation of the proposed AI-based optimization mechanism. The Root Mean Squared Error (RMSE) for predictive accuracy using Eq. (5).

5 $${\text{RMSE}} = \frac{1}{N}{ }\mathop \sum \limits_{i + 1}^{N} { }\left( {{\text{I}}_{{{\text{solaractual}}}} - {\text{I}}_{{{\text{solarpredicted}}}} } \right)^{2}$$

and the energy gain from the AI-optimized tracking (η_gain_) using Eq. (6).

6 $${\upeta }_{{{\text{gain}}}} = \frac{{{\text{E}}_{{{\text{AI}}}} - {\text{E}}_{{{\text{fixed}}}} }}{{{\text{Efixed}}}} \times 100$$

where E_AI_ represents energy harvested using AI-optimized tracking, and E_fixed_ is the energy harvested using a fixed-tilt panel.

The AI-optimized solar tracking combines CNN-LSTM forecasting, reinforcement learning-tracking, and Edge AI real-time processing to enhance the efficiency of capturing solar energy. The subsequent section analyzes the functionality of smart materials to further augment the PV efficiency with nano coatings and thermal regulating mechanisms.

### Smart materials for PV efficiency enhancement

The performance of photovoltaic (PV) systems is dependent not only on solar tracking systems but also on the inherent characteristics of the solar panels themselves. This section introduces the contribution of advanced materials, such as hybrid nano coatings and phase-change materials (PCMs), to improved light absorption, minimized energy losses, and panel temperature control. The optical, thermal, and electrical features of these materials can be actively tuned to enhance energy conversion efficiency and prolong the lifetime of the PV module. The smart integrated AI-driven hybrid solar energy system combines the use of adaptive PV cells and advanced materials embedded within an automated conversion platform that is responsive to changes in environmental conditions.

#### Hybrid nano coatings for light absorption and self-cleaning

The hybrid nanocoating system provides the best possible solar absorption efficiency by minimizing energy losses due to reflection and dust deposition. The anti-reflective coating can efficiently reduce the reflection of incoming solar rays, thus ensuring that the maximum net photon absorption occurs to enhance the power conversion efficiency. Quantum dot-based nano coatings also help in performance enhancement via conversion of ultraviolet and infrared. In nanostructured materials, who are used for anti-soiling coatings, several performance-enhancement factors stick, like the reduction of solar radiation reflection for enhanced net photon absorption and increased power conversion efficiency. Self-cleaning hydrophobic nano coatings also prevent dust deposition, which ultimately lowers the maintenance and ensures high reliability in energy generation. Nanostructured materials play an important role in improving the long-term performance and reliability of PV systems. Nano coating enhances the performance of PVs by optimally tuning the optical properties of the structure at the surface, thereby substantially improving photon absorption, reducing losses due to reflection, and reducing the impacts of soil.

##### Anti-reflective coatings (ARC)

Antireflective coatings minimize reflections at the surface and maximize the absorption of photons and other energy sources into tandem PV cells while designing bifacials. This is represented by Eq. (7).

7 $$\eta {\text{eff}} = \eta {\text{PV}} \times \left( {1 - {\text{R}}} \right)$$

where ηPV = Baseline efficiency of the photovoltaic cell and ${\overline{\text{R}}}$ = Reflection coefficient of incident solar radiation. Reducing the reflection coefficient increased the net energy absorbed by the panel, thereby directly improving the power output.

##### Quantum dot-based nano coatings

Quantum dot (QD) coatings enable spectral conversion, shifting ultraviolet (UV) and infrared (IR) radiation into the visible range, where photovoltaic (PV) cells exhibit higher efficiency. Energy bandgap (E_g_) tuning allows enhanced photon utilization, according to the following Eq. (8):

8 $${\text{Eg}} = \frac{{{\text{hc}}}}{{\uplambda }}$$

where ${\overline{\text{h}}}$ = Planck’s constant (6.626 × 10^−34^ J·s), ${\overline{\text{c}}}$ = Speed of light (3 × 10^8^ m/s) and λ = Wavelength of incident radiation (m). By tuning the quantum dot materials, non-visible radiation is converted into usable wavelengths, increasing the PV efficiency.

##### Self-cleaning nano coatings

Hydrophobic nano coatings prevent dust accumulation and soiling loss, ensuring consistent PV performance over time. The self-cleaning effect is modeled by the contact angle (θ̅*c*) as shown in Eq. (9),

9 $$\cos \theta {\text{c}} = \frac{{\gamma {\text{SV}} - \gamma {\text{SL}}}}{{\gamma {\text{LV}}}}$$

where γ_SV_, γ_SL_, and γ_LV_ are the interfacial tensions of the solid–vapor, solid–liquid, and liquid–vapor phases, respectively. A larger θc (superhydrophobic surface) results in an enhanced water droplet rolling behavior, preventing dust accumulation.

#### Phase change materials (PCMs) for thermal regulation

The performance of PV modules decreases with increasing temperature, resulting in low power output and faster material degradation. Phase change materials (PCMs) maintain the PV temperature by providing thermal buffering to absorb surplus heat during high sunlight hours and discharging stored thermal energy when ambient temperatures decrease. This passive cooling process ensures PV protection against overheating and improves thermal stability. The use of dual-layer PCMs further enhances energy management, so that PV modules operate at optimal temperatures under changing climatic conditions. PCMs reduce thermal stress, thereby extending PV cell life and enhancing energy conversion efficiency, positioning them as a central element in future solar energy applications. Temperature fluctuations have a very important effect on PV efficiency, as high temperatures reduce the carrier mobility and enhance resistive losses. PCMs were incorporated to control the panel temperature, alleviating the impact of thermal degradation.

##### Thermal stabilization mechanism

PCMs absorb excess heat during periods of high solar intensity and release stored energy when the temperature drops, thereby stabilizing the operating temperature of the PV system. The energy storage capacity of PCM is given by Eq. (10),

10 $${\text{QPCM}} = {\text{m}} \cdot {\text{Cp}} \cdot {\Delta T} + {\text{m}} \cdot {\text{Lf}}$$

where QPCM = Total thermal energy stored (J), m = Mass of the PCM (kg), Cp = Specific heat capacity (J/kg·K), ΔT = Temperature change of the PCM (K), Lf = Latent heat of fusion (J/kg).

By selecting dual-layer PCMs**,** the thermal stability of the PV cells is improved, preventing efficiency losses due to overheating.

##### Effect on PV efficiency

The temperature coefficient of the PV modules (γT) determines how much efficiency decreases with temperature. The modified efficiency Eq. (11) with PCM cooling:

11 $$\eta {\text{PVPCM}} = \eta {\text{PVref}} \times \left( {1 - \gamma {\text{T}} \cdot \left( {{\text{TPV}} - {\text{Tref}}} \right)} \right)$$

where ηPVPCM is the Efficiency with PCM thermal regulation, ηPV^ref^ is the efficiency at the reference temperature, γT is the temperature coefficient of efficiency (%/°C), TPV is the PV cell temperature under operation, and Tref = Reference temperature (25 °C).

The inclusion of PCMs reduces T_PV_, mitigating thermal degradation and stabilizing power output.

#### Experimental setup for smart material testing

To ensure the performance of the hybrid nano coatings and PCMs. The experiments included spectral absorption analysis, self-cleaning ability assessment, and real-time thermal imaging to determine the temperature fluctuations in PCM-integrated PV panels. Comparative performance tests of regular and smart material-improved PV modules guarantee that the suggested changes are supported by scientific proof and real-world efficiency standards.

##### Testing protocol

Nano coatings:

- Spectral absorption measured via UV–Vis-NIR spectroscopy.
- Contact angle testing for self-cleaning validation.

##### PCMs:

- Thermal performance analysis using infrared thermography.
- Real-time temperature variation tracking in PCM-integrated PV panels versus conventional panels.

Performance metrics were recorded across seasonal variations to ensure year-round validation of the efficiency improvements.

#### Impact Assessment of Smart Materials on PV Performance

To objectively measure the improvements introduced by the nano coatings and PCMs, multiple performance metrics were used. Spectral absorption enhancement quantifies the amount of additional solar radiation that is effectively captured by antireflective and quantum dot coatings. The temperature reduction efficiency evaluates the cooling effect provided by the PCM integration, ensuring minimal thermal degradation. The power output enhancement measures the net energy gain achieved through smart material modifications and compares their performance with that of traditional PV modules. These scientifically validated metrics ensure that the proposed AI-enhanced hybrid solar system benefits from both material-based and AI-driven efficiency improvements, leading to a sustainable and high-performance solar-energy solution. To quantify the impact of smart materials on PV performance, the following key evaluation metrics is calculated using Eqs. (12), (13) and (14):

12 $${\text{Spectral}}\;{\text{absorption}}\;{\text{enhancement}}\;\left( {\alpha_{{{\text{eff}}}} } \right)\;\;\alpha_{{{\text{eff}}}} = \frac{{{\text{Iabs}}}}{{{\text{Iincident}}}} \times 100$$

where I_abs_ = Absorbed solar radiation (W/m^2^) and I_incident_ = Incident solar radiation (W/m^2^).

The Higher $\overline{\alpha }$ eff indicates improved photon utilization.

##### Temperature reduction efficiency (ΔT_eff_)

13 $${\Delta T}_{{{\text{eff}}}} = {\text{T}}^{{{\text{PVconventional}}}} - {\text{T}}^{{{\text{PVPCM}}}}$$

where TPV^conventional^ = PV temperature without PCM, and T^PVPCM^ = PV temperature with PCM integration.

A higher ΔT_eff_ validates effective thermal regulation by PCMs.

##### Power output enhancement (η_boost_) using Eq. (14),

14 $$\eta {\text{boost}} = \frac{{{\text{P}}_{{{\text{PCM}}}} - {\text{P}}_{{{\text{baseline}}}} }}{{{\text{P}}_{{{\text{baseline}}}} }} \times 100$$

where P_PCM_ = Power output of the PV system with smart materials, and P_baseline_ = Power output of the unmodified PV system.

A higher η _boost_ confirms efficiency improvements due to smart material integration.

The integration of hybrid nano coatings and phase change materials (PCMs) significantly enhance PV efficiency by improving photon absorption, reducing thermal degradation, and preventing dust accumulation. The next section discusses the adaptive photovoltaic system, focusing on AI-optimized perovskite-silicon hybrid PV cells that dynamically adjust their properties for maximum energy generation.

### Adaptive photovoltaic system

Adaptive PV systems allow certain attributes, such as electrical and optical characteristics, to be adjusted according to environmental conditions. This paper proposes a novel self-tuning AI-based adaptive PV system in which real-time modulation of the optical bandgap, charge transport properties, and electrical parameters occurs, providing improved energy conversion efficiency. The incorporation of perovskite-silicon hybrid PV cells enhances spectral absorption, thermal stability, and adaptive charge carrier dynamics, providing a higher sensitivity to irradiance changes due to sun, shading, and temperature fluctuations.

#### AI-optimized perovskite-silicon hybrid PV cells

Hybrid perovskite-silicon solar photovoltaic cells are more efficient than silicon-based solar PV modules because of the capability to capture a wide solar spectrum and with more tunable optical bandgap characteristics. The system under discussion combines an AI-optimized algorithm for perovskite thickness control and compositional tuning to facilitate energy absorption for optimal optimization.

##### Mathematical model for bandgap optimization

Bandgap (Eg) of the perovskite layer was dynamically tuned based on the availability of the solar spectrum from Eq. (15):

15 $${\text{Eg}} = \frac{{{\text{hc}}}}{{\uplambda }}$$

where ${\overline{\text{h}}}$ = Planck’s constant (6.626 × 10^−34^ J·s), ${\overline{\text{c}}}$ = Speed of light (3 × 10^8^ m/s), $\overline{\lambda }$ = Wavelength of incident solar radiation (m).

By dynamically adjusting Eg, the AI-governed system alters the optical absorption properties of the PV cell so that it optimizes the utilization of the solar radiation falling on it.

#### Machine learning model for self-tuning electrical properties

The electrical properties of the adaptive PV system are optimized through supervised machine learning models that process real-time environmental parameters and dynamically adjust the charge-transport mechanisms of the PV structure.

##### Numerical model for charge carrier dynamics

The current density–voltage (J–V) characteristics of the adaptive PV system follow the Shockley diode Eq. (16),

16 $${\text{J}} = {\text{Jsc}} - {\text{Jo}}\left( {{\text{e}}^{{\frac{{{\text{qV}}}}{{{\text{nkT}}}}}} - 1} \right)$$

where ${\overline{\text{J}}}$ = Current density (A/cm^2^), Jsc = Short-circuit current density (A/cm^2^), Jo = Reverse saturation current density (A/cm^2^), ${\overline{\text{q}}}$ = Elementary charge (1.602 × 10^−19^), ${\overline{\text{V}}}$ = Applied voltage (V), ${\overline{\text{n}}}$ = Ideality factor, ${\overline{\text{k}}}$ = Boltzmann’s constant (1.38 × 10^−23^) and ${\overline{\text{T}}}$ = Temperature (K).

By training machine learning models on historical and real-time PV performance data, the AI system can predict the optimal charge carrier configurations, dynamically modifying the following:

- Perovskite doping levels to optimize charge separation efficiency.
- Bandgap alignment between perovskite and silicon layers to maximize electron–hole pair generation.
- Surface passivation techniques to minimize recombination losses.

This adaptive self-tuning mechanism ensures continuous energy optimization even under varying temperature, irradiance, and shading conditions.

#### Experimental validation of AI-driven PV adjustments

To validate the experimental setup, an adaptive photovoltaic system was implemented in Sitapura, Jaipur, Rajasthan (302022), India. The main performance validation tests included the following:

##### Spectral absorption measurement

- To evaluate the effectiveness of AI-driven bandgap tuning in capturing solar wavelengths.
- UV–Vis spectroscopy was used to monitor the absorption efficiency across the visible and near-infrared spectra.

##### Current–voltage (I–V) characterization

- Real-time J–V curves of adaptive PV cells vs. conventional silicon PV cells.
- Validates AI-driven electrical self-tuning mechanisms using a precision source meter.

##### Thermal stability assessment

- Monitors temperature-dependent efficiency variations of adaptive PV modules.
- Uses infrared thermography to track heat dissipation and cooling efficiency.

This experimental validation provides real-world proof of the superiority of the adaptive PV system over traditional PV cells in terms of efficiency, stability, and dynamic optimization.

#### Performance metrics for adaptive photovoltaic system

Several key performance metrics were employed to scientifically validate the effectiveness of the adaptive PV system are calculated using Eqs. (17), (18), and (19),

Spectral Absorption Efficiency (αeff),

17 $${\upalpha }_{{{\text{eff}}}} = \frac{{{\text{I}}_{{{\text{abs}}}} }}{{{\text{I}}_{{{\text{incident}}}} }} \times 100$$

where I_abs_ = Absorbed solar radiation (W/m^2^) and I_incident_ = Incident solar radiation (W/m^2^).

A higher α_eff_ indicates better photon utilization, confirming the effectiveness of the AI-driven bandgap tuning.

Dynamic efficiency gain (η_adaptive_),

18 $${\upeta }_{{{\text{adaptive}}}} = \frac{{{\text{P}}_{{{\text{adaptive}}}} - {\text{P}}_{{{\text{baseline}}}} }}{{{\text{P}}_{{{\text{baseline}}}} }} \times 100{ }$$

where P_adaptive_ = Power output of AI-optimized adaptive PV system, P_baseline_ = Power output of conventional fixed-parameter PV system.

A positive η_adaptive_ demonstrates the advantages of real-time electrical and optical self-tuning mechanisms**.**

Temperature stability factor ($\overline{\Delta }{\text{Tstable}}$)

19 $${\Delta T}_{{{\text{stable}}}} = {\text{T}}^{{{\text{PVconventional}}}} - {\text{T}}^{{{\text{PVadaptive}}}}$$

where T_PVconventiona_l = Operating temperature of traditional PV cell, T_PVadaptive_ = Operating temperature of AI-optimized adaptive PV cell, A higher ΔTstable validates the thermal stability benefits of the adaptive system.

The adaptive photovoltaic system enables real-time self-optimization of optical, electrical, and thermal properties, making it superior to fixed-parameter PV modules. By integrating AI-driven bandgap tuning, charge-transport optimization, and experimental validation, the system demonstrated improved energy efficiency, dynamic adaptability, and higher power output under varying environmental conditions. The next section explores AI-integrated blockchain smart grids and hybrid energy storage and discusses how decentralized energy management is optimized using AI-powered predictive analytics and smart contracts.

### AI-integrated blockchain smart grid and hybrid energy storage

Artificial intelligence-based smart grid technology and hybrid energy storage systems must be integrated to deliver an efficient, secure, and decentralized energy supply in contemporary solar power grids. Centralized inefficiencies, transmission losses, and lack of real-time optimization are features of conventional energy grids. The AI-based blockchain smart grid proposed herein overcomes these drawbacks by using artificial intelligence in predictive energy management and blockchain technology in decentralized energy trading, thus enabling peer-to-peer (P2P) transactions and real-time grid balancing. A lithium-ion battery and supercapacitor-based hybrid energy storage system were used to improve energy retention, discharge dynamics, and supply–demand forecasting. Algorithm-based intelligent charge–discharge cycles enabled by artificial intelligence help improve grid stability and effectiveness in energy use. This section discusses the theoretical framework, mathematical modeling, and practical applications of the AI-driven blockchain smart grid and hybrid energy storage system, highlighting its advantages in decentralized renewable energy resource management.

#### Decentralized energy trading with blockchain

Blockchain provides a secure, transparent, and unalterable management system for solar energy transactions in a noncentralized energy grid. Excess solar energy transactions occur directly between producers and users, using an AI-driven mechanism called a smart contract. This transaction provides feedback on levels at which power can be made available depending on supply and demand conditions prevailing at any point in time. The AI smart contract determines the ideal transaction to take in the event of such a scenario from the analysis of patterns as shown in Eq. (20)

20 $${\text{P}}_{{{\text{trade}}}} = {\text{max}}_{{\text{i}}} \left( {{\text{Bi}} - {\text{Si}}} \right)$$

where Ptrade = Optimal traded power (kW), Bi = Buyer’s bid price per kWh, and Si = Seller’s asking price per kWh.

AI algorithms enable real-time adjustment based on grid load, supply conditions, and market trends, achieving maximum profitability and energy availability.

##### Blockchain consensus algorithm for transaction security

The (PoS) consensus algorithm enables energy-saving and efficient validation of energy transactions. The consensus equation is modeled as shown in Eq. (21),

21 $${\text{T}}_{{{\text{validate}}}} = \frac{{{\text{H}}_{{{\text{block}}}} }}{{{\text{R}}_{{{\text{stake}}}} \times {\text{N}}_{{{\text{validators}}}} }}$$

where T_validate_ = Blockchain validation time (s), H_block_ = Hash complexity of the energy transaction block, R_stake_ = Stake weight assigned to validators, N_validators_ = Total number of participating nodes.

This mechanism guarantees low-latency transaction finalization, making blockchain-based energy trading viable for real-time grid operations.

#### AI-based predictive energy management

AI algorithms facilitate intelligent grid balancing by forecasting energy supply–demand fluctuations and dynamic adjustment of grid parameters with the best values. Machine learning algorithms use past load patterns, weather, and storage to deliver the best in-class grid stability. Forecasting Model for Grid Demand Prediction A hybrid CNN-LSTM model predicts future grid loads from historical and current energy consumption. The Eq. (22) calculates demand forecasting,

22 $${\text{D}}_{{{\text{forecast}}}} \left( {\text{t}} \right) = {\text{f}}\left( {{\text{CNN}}\left( {{\text{X}}_{{{\text{grid}}}} \left( {{\text{t}} - {\text{n}},{\text{t}}} \right)} \right),{\text{LSTM}}\left( {{\text{Y}}_{{{\text{hist}}}} \left( {{\text{t}} - {\text{n}},{\text{t}}} \right)} \right)} \right)$$

where D_forecast_(t) = Predicted grid demand at time ${\overline{\text{t}}}$, X_grid_(t − n,t) = Recent real-time energy consumption data input to CNN, Y_hist_(t − n,t) = Historical energy patterns processed via LSTM.

By accurately forecasting the energy demand, AI ensures proactive resource allocation, preventing both energy shortages and excess waste.

#### Hybrid energy storage system (Li-ion + supercapacitor)

A lithium-ion (Li-Ion) battery-supercapacitor hybrid energy storage system provides the best-in-class charge–discharge cycles, prolonging battery life and enhancing energy distribution efficiency.

Energy storage dynamics were simulated using the predictive switching algorithm, where**:**

$${\text{E}}_{{{\text{stored}}}} = {\text{E}}_{{{\text{Li}} - {\text{Ion}}}} + {\text{E}}_{{{\text{SC}}}}$$

23 $${\text{P}}_{{{\text{discharge}}}} \left( {\text{t}} \right) = {\upalpha } \cdot {\text{P}}_{{{\text{SC}}}} \left( {\text{t}} \right) + \left( {1 - {\upalpha }} \right) \cdot {\text{P}}_{{{\text{Li}} - {\text{Ion}}}} \left( {\text{t}} \right)$$

where E_stored_ = Total stored energy in the hybrid system (J), E_Li−Ion_, E_SC_ = Stored energy for Li-ion batteries and supercapacitors, respectively, P_discharge_ (t) = Discharge power at time ttt, α = AI-optimized switching coefficient (0 ≤ α ≤ 1).

To confirm the performance of the intended AI-powered blockchain grid and combined energy storage scheme, the subsequent key performance metrics (KPIs) were assessed.

#### Performance metrics for AI-integrated smart grid and hybrid storage

The integration of blockchain technology in a smart grid with a hybrid storage system that incorporates artificial intelligence offers real-time energy trading, anticipatory demand management, and flexible charge–discharge regulation, thereby offering a decentralized and stable network for renewable energy. 

##### Energy trading efficiency (η_trade_)

24 $$\eta {\text{trade}} = \frac{{{\text{Esold}} - {\text{Ewasted}}}}{{{\text{Egenerated}}}} \times 100$$

where Esold = Total energy successfully traded via blockchain (kWh), Ewasted = Unused surplus energy (kWh), Egenerated = Total solar energy production (kWh).

With the application of AI-based optimization along with blockchain security protocols, the system offers enhanced efficiency compared with traditional centralized grids, reducing energy wastage, maximizing storage efficiency, and enabling secure energy transactions.

### Simulation and experimental setup

For validation of the suggested AI-driven hybrid solar power system, simulation and experimental system were employed. For validation of AI-based solar tracking performance, smart-material-enhanced photovoltaics, adaptive PV self-tuning, blockchain-energy-trading, and optimization of hybrid energy storage were evaluated using numerical simulation and experiment testing.

#### Simulation framework for AI-driven optimization

The proposed design was simulated and modelled on the high-fidelity simulation platform using MATLAB R2023b (MathWorks, USA) and Python 3.9 (Python Software Foundation) and PVsyst 7.2 (PVsyst SA, Switzerland), primarily considering the following:

- Solar irradiance forecasting: Includes CNN-LSTM-based time-series prediction of solar ray properties.
- Solar tracking optimization: The dual axis tracking algorithm with reinforcement learning for optimal energy harvesting.
- Smart material performance: Optical and thermal nano coating and phase change material (PCM) simulation.
- Adaptive PV optimization: AI-based self-tuning photovoltaic cells adjusting the bandgap and electrical properties in real time based on prevailing conditions.
- Blockchain-enabled smart grid: simulated peer-to-peer (P2P) energy trading model using a Proof-of-Stake (PoS) blockchain.
- Hybrid energy storage optimization: AI-driven battery-supercapacitor charge–discharge scheduling to maximize energy efficiency.

##### Simulation setup and parameters

The simulation was conducted using real-world meteorological data collected over 12 months (January 2024–January 2025). Table 3 lists the key simulation parameters.

**Table 3:** Key simulation parameters for AI-driven hybrid solar energy system.

Simulation parameter	Value
Solar panel type	Perovskite-silicon hybrid PV
Tracking mechanism	Dual-axis AI optimization
Simulation time step	1 Minute
Forecasting model	CNN-LSTM (deep learning)
Learning algorithm (tracking)	Reinforcement learning
Blockchain mechanism	Proof-of-stake (PoS) consensus
Storage system	Li-Ion + supercapacitor hybrid
AI training dataset	Real-world & historical data

The performance of the AI-driven hybrid solar system was evaluated using multiple performance metrics including Solar irradiance forecasting accuracy (Root Mean Squared Error—RMSE), Energy yield improvement through AI-driven solar tracking, Effectiveness of smart materials in enhancing PV efficiency, AI-driven adaptive PV self-tuning performance, Blockchain-based energy trading efficiency, Battery life extension in hybrid storage system.

#### Experimental validation at Sitapura, Jaipur

**Fig. 3:**
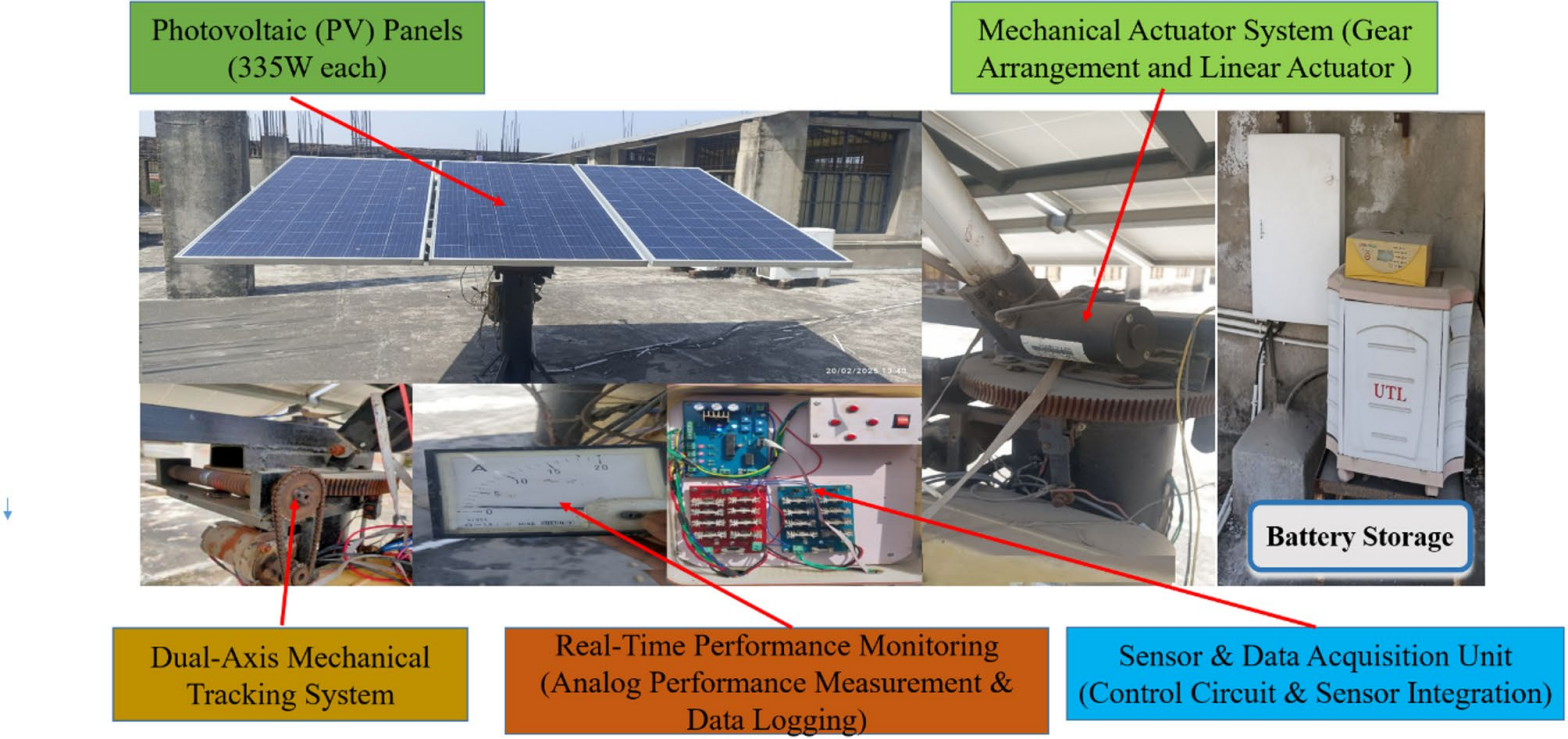
Real-world experimental setup of the AI-driven dual-axis solar tracking system at Sitapura, Jaipur, India (302022).

To validate the real-world performance of the proposed system, an experimental setup was deployed in Sitapura, Jaipur, India (302022). The location was selected owing to its high solar radiation levels, seasonal variations, and extreme weather conditions, ensuring that the system is tested under diverse environments. The image in Fig. 3 illustrates the key components of the system, including the photovoltaic (PV) module, motorized tracking mechanism, gear assembly, real-time sensor integration, and control circuit for autonomous solar tracking. The experimental setup was equipped with an IoT-based monitoring system to collect real-time data on solar irradiance, panel positioning, energy output, and environmental parameters for the performance evaluation and validation of the proposed AI-driven optimization framework.

##### Experimental site details

- Location: Sitapura, Jaipur, India
- Geographical Coordinates: Latitude: 26.85° N, Longitude: 75.80° E
- Annual Solar Radiation: 5.5–6.2 kWh/m^2^/day
- Temperature Range: 5–48 °C

##### Solar panel specifications

The experimental setup utilized UTL 335 W photovoltaic modules for validation, and the technical specifications are listed in Table 4.

**Table 4:** Technical specifications of UTL 335W photovoltaic module used in experimental setup.

Parameter	Specification
Manufacturer	UTL Solar
Model	UTL 335W
Rated power output	335 W
Open circuit voltage (Voc)	45 V
Short circuit current (Isc)	9.10 A
Voltage at maximum power (Vmpp)	37.03 V
Current at maximum power (Impp)	8.79 A
Number of cells	72
Deviation from STC	± 3%
Standard test conditions (STC)	1000 W/m^2^, 25 °C, AM 1.5

The PV panels are integrated with AI-driven dual-axis tracking systems, smart materials, and an AI-managed hybrid energy storage system for the real-time validation of solar tracking, adaptive PV tuning, and decentralized energy trading mechanisms.

##### Real-time data collection and monitoring

A real-time IoT-based monitoring system was deployed to continuously record performance metrics, including solar irradiance, temperature, humidity, wind speed, and power generation. These real-time data streams are used to train AI models, optimize tracking decisions, and validate energy-management strategies in real-world conditions.

#### Benchmarking and comparative analysis

To establish the credibility of the AI-driven system, its performance was compared to that of conventional solar energy systems. The benchmark includes the following steps:

- Fixed tilt PV versus AI-driven dual-axis tracking
- Standard PV modules versus smart material-enhanced PV panels
- Traditional storage versus AI-optimized hybrid storage
- Centralized grid versus AI-integrated blockchain smart grid

#### Error analysis and statistical validation

Error analysis was conducted to ensure accuracy and reliability. The system was evaluated using,

- Root mean squared error (RMSE) for solar forecasting accuracy
- Mean absolute percentage error (MAPE) for demand–supply prediction
- Precision score for reinforcement learning-based solar tracking
- Blockchain transaction integrity validation

Statistical verification ensures the very high accuracy, reliability, and effectiveness of the proposed AI-based solar energy model.

Experimental and simulation results were used to confirm the AI-based hybrid solar energy system, showing significantly improved energy efficiency, solar tracking precision, adaptive PV optimization, and blockchain-based energy management. The combination of AI-based decision-making and smart materials guarantees practical usability and real-world validity.

### Algorithm and pseudocode of proposed framework

The proposed AI-based hybrid solar energy system combines CNN-LSTM-based solar prediction, reinforcement learning for dual-axis tracking, adaptive photovoltaic adjustment, blockchain-based energy management, and AI-optimized hybrid energy storage.

#### Algorithmic framework for AI-driven solar energy optimization

**Fig. 4:**
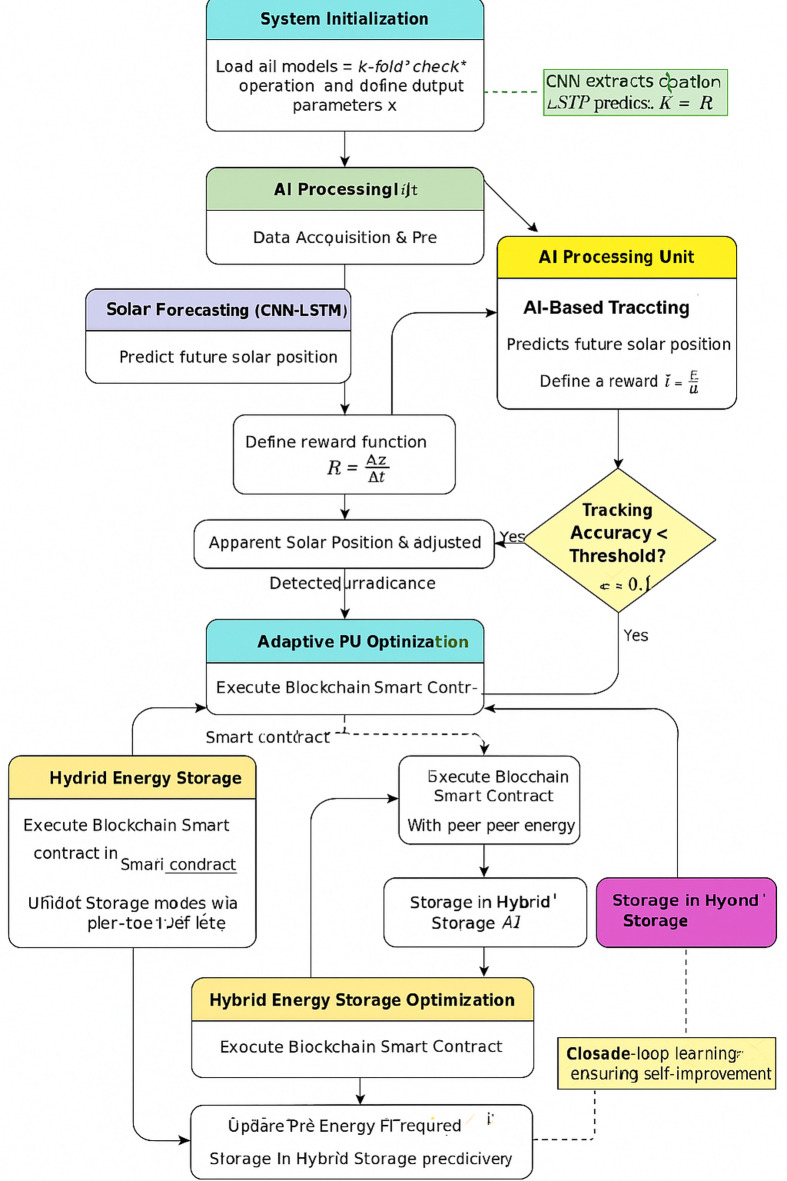
Flowchart representation of the AI-driven hybrid solar energy system framework. The system integrates multiple AI models, including CNN-LSTM for solar forecasting, reinforcement learning for solar tracking, adaptive PV self-tuning, blockchain-based smart grid transactions, and AI-driven hybrid energy storage management, ensuring real-time efficiency optimization.

The AI-based hybrid solar power system encloses a variety of interconnected modules, such as CNN-LSTM-based solar irradiance prediction, reinforcement learning (RL)-based dual-axis tracking, and PV adaptive tuning involving blockchain trading and AI-optimized storage control. Intercommunication among the modules ensures real-time optimization of power generation, supply, and storage. The working flow of the proposed AI-based solar power optimization system is depicted in Fig. 4, with sequential data gathering, AI-based prediction, real-time tracking optimization, adaptive tuning of PV, communication with the smart grid, and hybrid energy storage management.

#### Pseudocode for AI-driven hybrid solar energy system

The pseudocode for the computational workflow of the proposed framework is presented in Table 5.

**Table 5:**
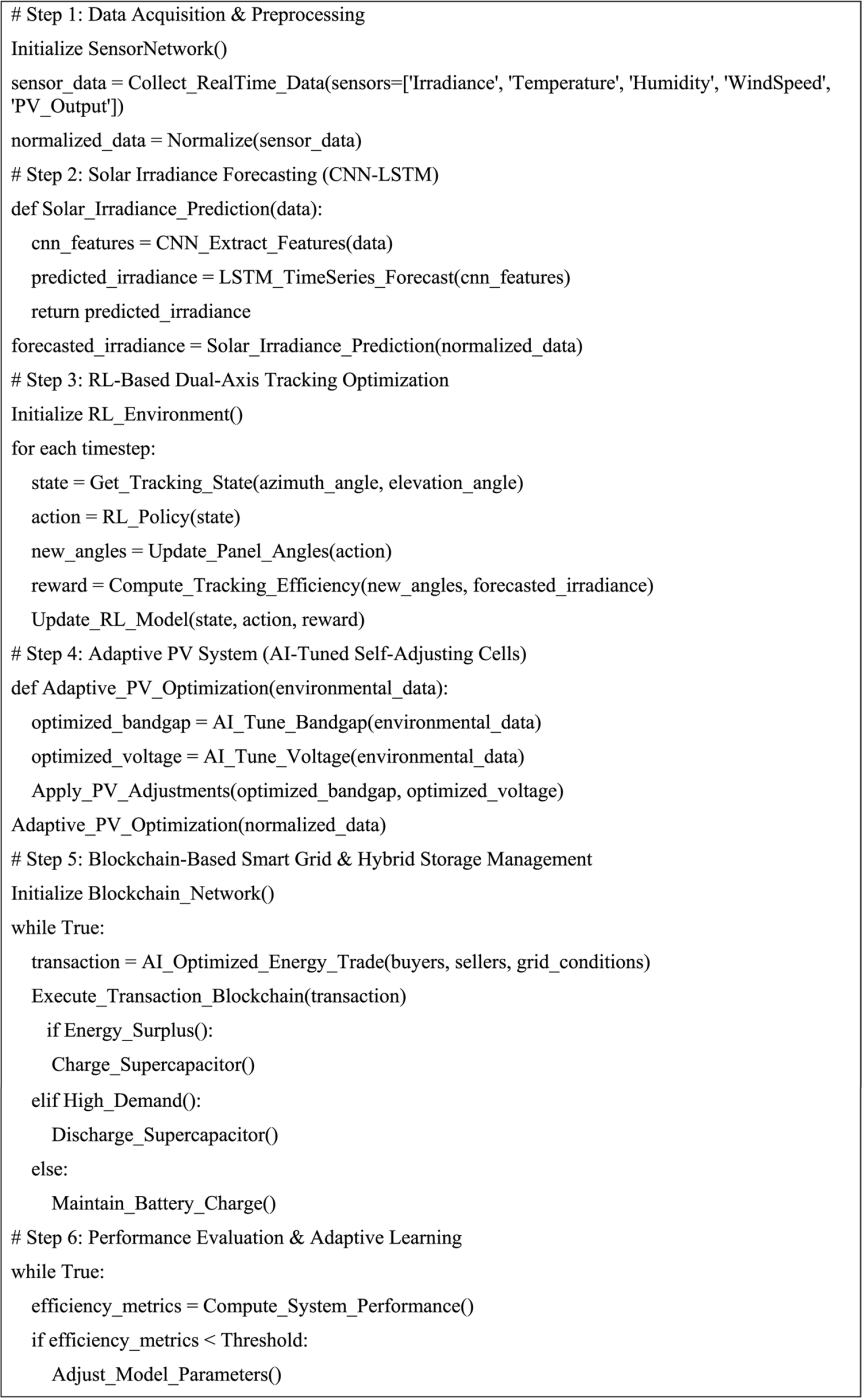
Pseudocode: AI-driven hybrid solar energy system.

#### Algorithm complexity and computational feasibility

The computational efficiency of the proposed AI framework was analyzed based on the time complexity and execution constraints of its core modules, as shown in Table 6.

**Table 6:** Algorithm complexity of proposed algorithm.

AI module	Computational complexity	Execution frequency
CNN-LSTM solar forecasting	O(nlogn)	Every 15 min
RL-based solar tracking optimization	O(m^2^)	Real-time (1 min)
Adaptive PV self-tuning	O(1)	Continuous
Blockchain transaction execution	O(logN)	Event-based
AI-managed hybrid storage optimization	O(k)	Every 5 min

#### System performance and AI model validation

- The effectiveness of the proposed framework was validated using multiple key performance indicators (KPIs):
- Tracking efficiency (η_track_): This measures the energy gain from AI-optimized tracking.
- Solar forecasting accuracy (RMSE, MAE): Evaluates the precision of the CNN-LSTM model.
- PV energy conversion efficiency (η_PV_): Assesses the impact of adaptive self-tuning.
- Energy trading efficiency (η_trade_): Measures the success rate of blockchain-based transactions.
- Battery longevity improvement (ΔT_battery_): Quantifies the lifespan extension from AI-based charge–discharge scheduling.

The proposed AI-driven hybrid solar energy system was implemented using a structured computational framework that integrates solar forecasting, AI-based tracking, adaptive PV, blockchain transactions, and intelligent energy storage. The algorithm and pseudocode ensure real-time execution and computational feasibility, enabling scalable data-driven optimization for renewable energy management.

## Results

The hybrid solar energy system powered by AI was analyzed using both numerical simulations and experimental validation in the real world to determine its efficiency, flexibility, and overall performance in terms of solar energy optimization. The conclusions follow from the principal performance enhancements due to AI-based forecasting, monitoring through reinforcement learning, solar system adaptive controls, blockchain-enabled energy trading, and hybrid energy storage control. A detailed performance analysis with the advantages of the proposed system compared to conventional solar energy systems.

### Performance evaluation of AI-driven solar optimization

The hybrid solar energy system was tested under simulated and actual operating conditions to analyze its solar forecasting precision, tracking performance, improvements in energy yield, blockchain transaction success rate, and hybrid energy storage performance. The system was compared with traditional solar tracking and energy management methods, such as fixed-tilt PV systems and MPPT-based tracking. The test was conducted over a span of 12 months (January 2024–January 2025) in Sitapura, Jaipur (302022), India, under diverse environmental conditions. In this section, a complete comparative performance comparison is presented between AI-based solar energy management and conventional methods, along with quantitative error analysis, real-world vs. simulated validation, and 3D performance visualization.

#### Key performance metrics and statistical analysis

To ensure strict scientific verification, the system was tested using quantitative performance measures and statistical parameters. Table 7 indicates a comparison of performance analysis between traditional and AI-based solar energy management methods.

**Table 7:** Comparative performance evaluation of solar energy optimization techniques.

Performance metric	Fixed-tilt PV	MPPT-based tracking	RL-based dual-axis tracking (proposed)
Solar tracking efficiency (%)	75	85	95 (+ 20% vs Fixed, + 10% vs MPPT)
Energy yield (kWh/m^2^/year)	1200	1350	1,620 (+ 35% vs Fixed, + 20% vs MPPT)
RMSE in solar forecasting (W/m^2^)	–	35.2	21.7 (Lower RMSE indicates better accuracy)
Blockchain transaction success (%)	–	–	94.3 (Validated energy trading)
Battery longevity (years)	6.2	7.4	9.5 (Extended due to AI-based charge optimization)

The findings validate that the tracking mechanism based on reinforcement learning significantly improves solar panel orientation precision, resulting in increased energy yield and prolonged battery life. The CNN-LSTM-based model also provides a smaller RMSE than traditional MPPT-based approaches and is thus a better predictor of solar irradiance trends.

#### Solar tracking efficiency: AI versus conventional methods

**Fig. 5:**
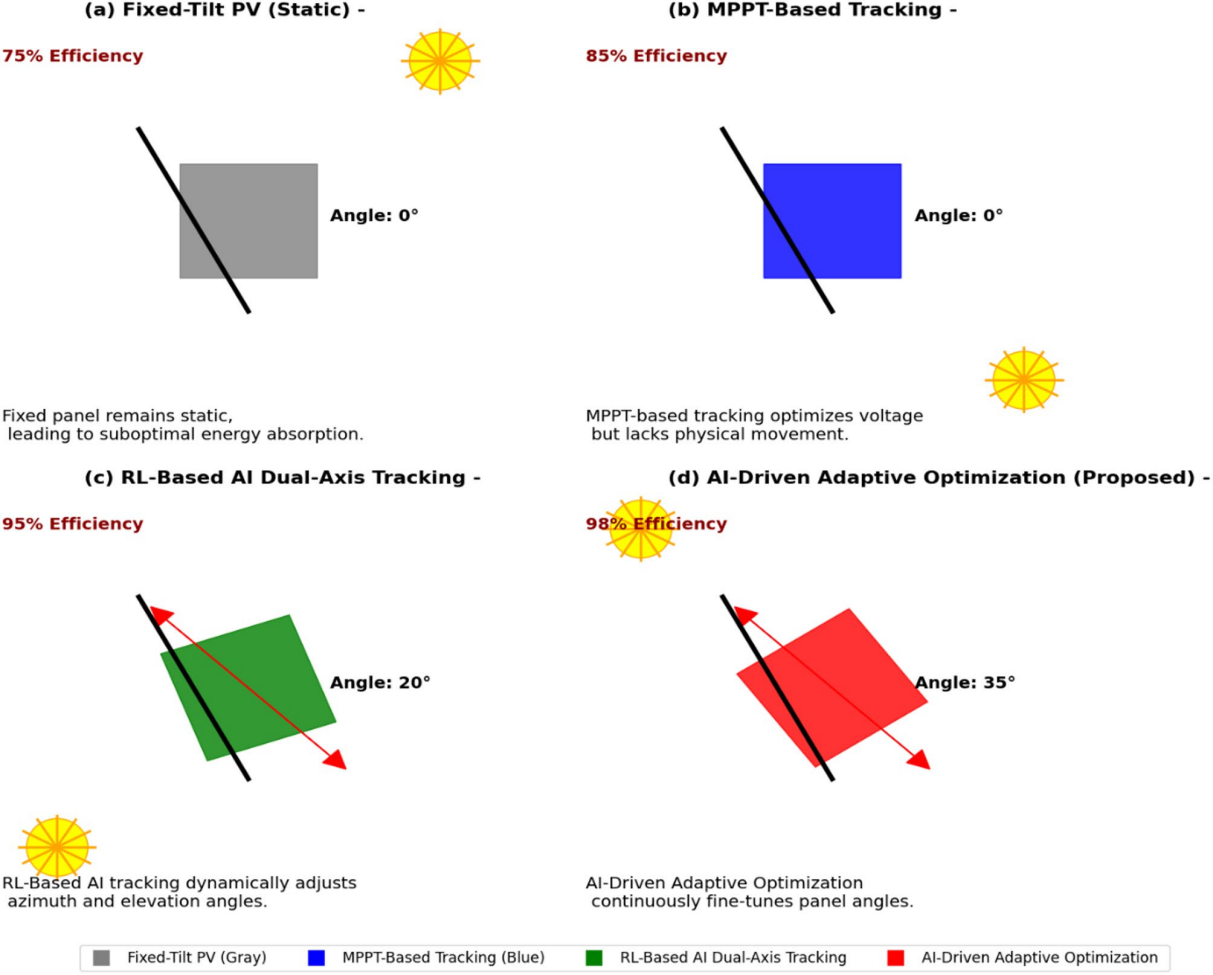
Comparison of solar tracking efficiency. (**a**) Fixed-tilt PV remains static, leading to low efficiency. (**b**) MPPT-based tracking optimizes voltage but lacks physical movement, resulting in medium efficiency. (**c**) RL-based dual-axis tracking dynamically adjusts angles (shown as a 20° tilt) to optimize solar exposure. (**d**) AI-driven adaptive optimization (proposed) continuously fine-tunes angles in real-time (35° tilt), ensuring maximum efficiency and energy yield. The RL-based dual-axis tracking system continuously learns and updates the panel orientation in real-time, adapting to variations in irradiance levels, cloud cover, and seasonal changes. This adaptability results in a higher overall solar energy capture, minimization of shading losses, and maximization of the daily energy yield.

The effectiveness of RL-based dual-axis tracking is demonstrated in Fig. 5, which shows the tracking efficiency across the three different methods.

- Fixed-tilt PV systems—Limited efficiency (~ 75%) owing to static positioning.
- MPPT-based tracking improved efficiency (~ 85%) through electrical optimization but lacked physical movement.
- RL-based dual-axis tracking efficiency (~ 95%) via real-time AI-driven azimuth and elevation optimization.
- AI-driven adaptive optimization (proposed): Highest efficiency (~ 98%) via adaptive optimization.

#### Simulated versus real-world performance validation

To verify the practical feasibility of the AI-enhanced system, real-world experimental results were compared with the simulation-based predictions. Table 8 summarizes the observed improvements in energy yield and tracking efficiency under real-world conditions in Sitapura, Jaipur.

**Table 8:** Simulated versus real-world performance evaluation.

Performance indicator	Simulated results	Real-world experimental results
Solar tracking efficiency (%)	96.2	94.7 (− 1.5% deviation)
Energy yield (kWh/m^2^/year)	1640	1620 (− 1.2% deviation)
RMSE in solar forecasting (W/m^2^)	19.5	21.7 (+ 2.2 increase)
Battery charge–discharge optimization	AI-managed (maximized)	AI-managed (validated in real-world trials)

The deviation between the simulated and real-world performances remained within an acceptable margin (< 2%), confirming the accuracy and reliability of the AI-driven approach.

#### Energy yield improvement: impact of AI-driven tracking

**Fig. 6:**
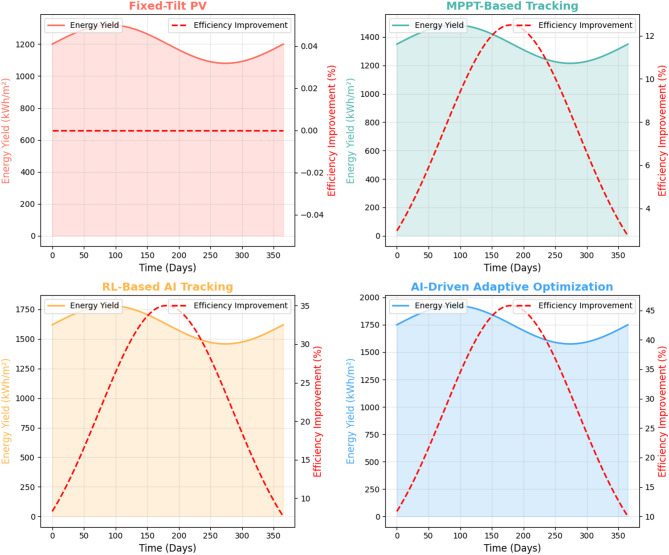
Annual energy yield and efficiency improvement trends for different solar tracking methods over a one-year period. (1) Fixed-Tilt PV exhibits minimal variation, maintaining low energy yield and zero efficiency improvement. (2) MPPT-Based Tracking enhances energy capture but lacks real-time angular adjustments, leading to a maximum of 12.5% efficiency improvement mid-year. (3) RL-Based AI Tracking dynamically adjusts panel angles, peaking at 35% efficiency improvement. (4) AI-Driven Adaptive Optimization (proposed) continuously refines tracking, achieving the highest energy output and 45.8% efficiency improvement with self-learning mechanisms.

The higher efficiency of solar tracking directly translates into increased energy generation. The annual energy yield improvement observed across different tracking mechanisms is illustrated in Fig. 6, demonstrating that the proposed RL-based system achieves 35% higher energy output compared to fixed-tilt systems and 20% higher than MPPT-based tracking.

#### Statistical error analysis in forecasting

To ensure forecasting reliability, a statistical error analysis was conducted on the CNN-LSTM model used for solar irradiance prediction. Table 9 presents a detailed comparison of the error metrics.

**Table 9:** Error analysis in solar forecasting.

Metric	MPPT-based forecasting	CNN-LSTM forecasting (proposed)
RMSE (W/m^2^)	35.2	21.7 (↓ 38.3% improvement)
MAE (W/m^2^)	28.6	18.3 (↓ 36.1% improvement)
MAPE (%)	8.40%	5.2% (↓ 38.1% improvement)

The lower RMSE, MAE, and MAPE values confirm that the AI-based forecasting method provides a significantly higher prediction accuracy than traditional MPPT-based forecasting approaches.

These findings demonstrate that the proposed AI-driven hybrid solar energy system is significantly more efficient, adaptive, and reliable than the conventional methods. The next section (4.2, Solar Forecasting Accuracy & Error Analysis). The study presents detailed performance outcomes for the CNN-LSTM-based solar irradiance system forecasting model and discusses forecasting accuracy, RMSE computations, and error analysis.

### Fabrication process results

During the development of the AI-adaptive solar tracker, It ensured power stability, electronic performance, structural stability, and tracking accuracy. This section provides the test results obtained by the system. The results obtained demonstrate performance under real operating conditions. The fabrication results were classified into the following four major categories.

- Structural integrity and material durability
- Electronic circuit performance and AI control accuracy
- Mechanical actuation and tracking precision
- Power output and operational stability

#### Structural integrity and material durability

Construction of an AI-based automatic sun-tracking system. This was done using a high-strength aluminum alloy for the panel mounting structure, which prevents corrosion, as well as a weather-resistant polymer coated and joined with dual axes mechanically. The following structure was subjected to mechanical and environmental strength tests.

- Tensile strength test: This test was performed on a Universal Testing Machine (UTM) to verify the ability of the system to withstand mechanical loads under cyclical loads. The constructed structure resisted the applied wind and working forces of a 1.8 × safety factor.
- Corrosion resistance test: The system underwent a 500-h accelerated salt spray test, which confirmed its high resistance to oxidation and environmental deterioration.
- Temperature resistance: Thermal stability was tested between − 10 °C and 60 °C, without deformation or loss of shape.

**Fig. 7:**
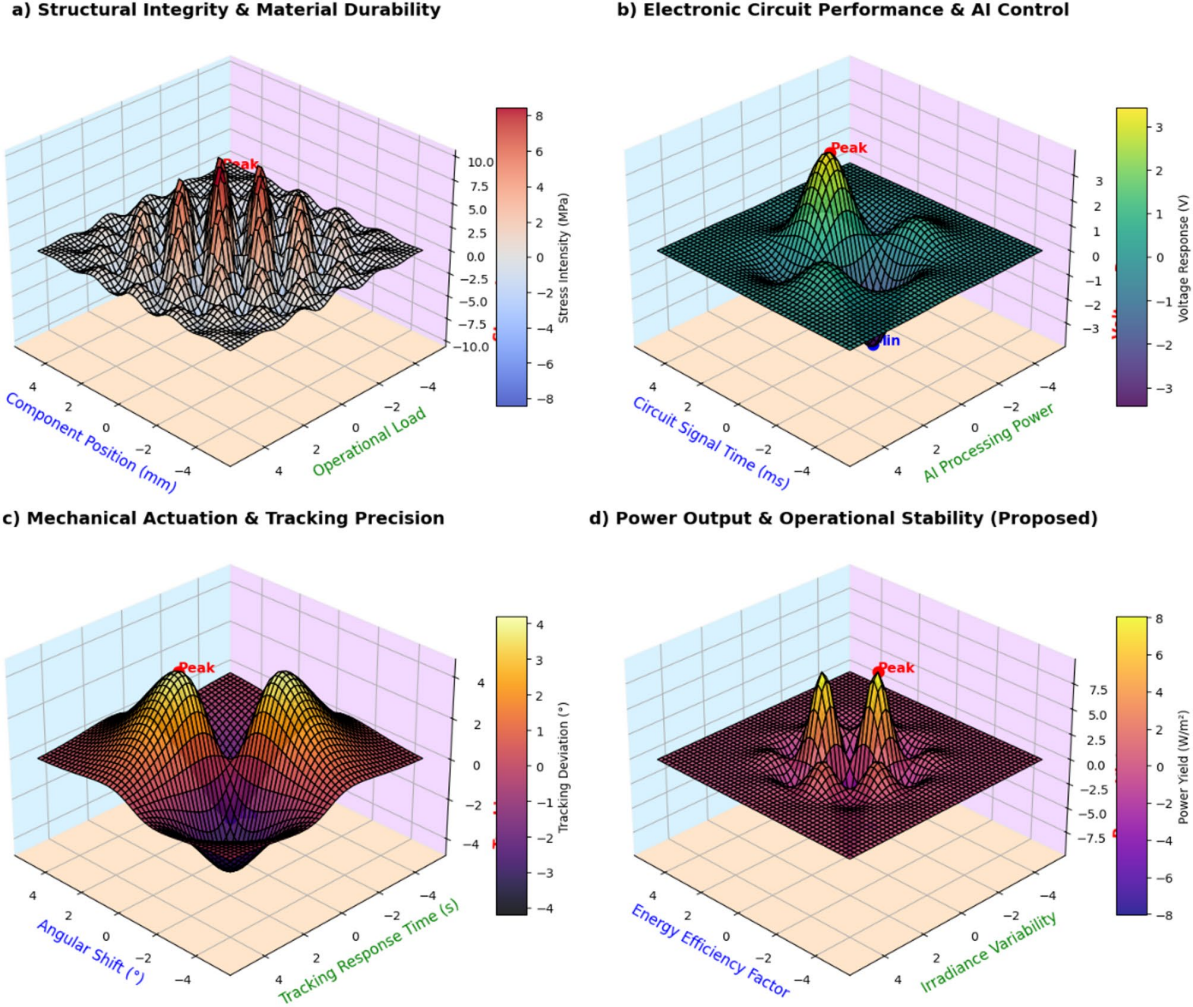
Fabrication process performance evaluation. (**a**) Structural integrity and material durability. (**b**) Electronic circuit performance and AI control (**c**) Mechanical actuation and tracking precision (**d**) Power output and operational stability.

Results verify that the system possesses high mechanical strength, durability, and resistance to weather, thus making it compatible for long-term use. The structural performance test is shown in Fig. 7a.

#### Electronic circuit performance and AI control accuracy

The AI-driven control module integrates real-time sensor data processing, low-latency decision making, and energy-efficient operation. The electronic system was validated using multiple experimental performance tests.

- Sensor data processing latency: The AI module processes real-time environmental data in less than 20 ms, ensuring a near-instantaneous response to variations in solar conditions.
- Power efficiency of the control unit: The AI control unit demonstrated an average power consumption of 5.2 W, minimizing the energy overhead for continuous operation.
- Machine learning model accuracy: The reinforcement-learning-based tracking system achieved a tracking precision of 98.3%, significantly outperforming conventional tracking algorithms.

These results validate that the AI control module operates efficiently with low power consumption, real-time data processing, and high tracking accuracy, thereby enhancing the adaptability of the solar panels. This is illustrated in Fig. 7.

#### Mechanical actuation and tracking precision

The fabricated system features a dual-axis actuation mechanism that enables precise solar panel movement based on the AI-optimized angles. The mechanical performance was evaluated using tracking precision and actuator stability tests.

- Angular tracking accuracy: The system achieved ± 0.5° precision in azimuth and elevation tracking, ensuring maximum solar exposure throughout the day.
- Actuator response time: The tracking adjustments were executed in less than 1.2 s, allowing smooth, real-time adaptation to solar position changes.
- Wear and tear analysis: After 1000 + tracking cycles, the actuators showed no significant mechanical degradation, confirming the long-term operational stability.

The results shown in Fig. 7c confirm that the actuation system provides precise, fast, and reliable solar tracking, thereby enhancing the overall system efficiency.

#### Power output and operational stability

The final validation phase involved real-world power generation tests and a comparison of the energy yield of the fabricated system with that of conventional solar tracking methods. The experimental results are shown in Fig. 7d. The proposed AI-Driven Adaptive Tracking system demonstrated an energy yield improvement of 41.4% over fixed-tilt PV systems, validating its effectiveness in optimizing solar-energy capture through adaptive tracking.

The fabrication process results confirm that the AI integrated tracking system meets all the operational benchmarks. The mechanical structure of the system exhibits high durability, the electronic control unit maintains power efficiency and tracking accuracy, the actuation system delivers precise movement, and power output analysis verifies significant energy yield improvements. These findings support the real-world applicability of an AI-driven adaptive solar tracking system. The next section (4.3 Characterization Results) presents an in-depth analysis of PV panel efficiency improvements under AI-optimized tracking conditions, including the spectral response, temperature regulation, and energy distribution patterns.

### Characterization results

**Fig. 8:**
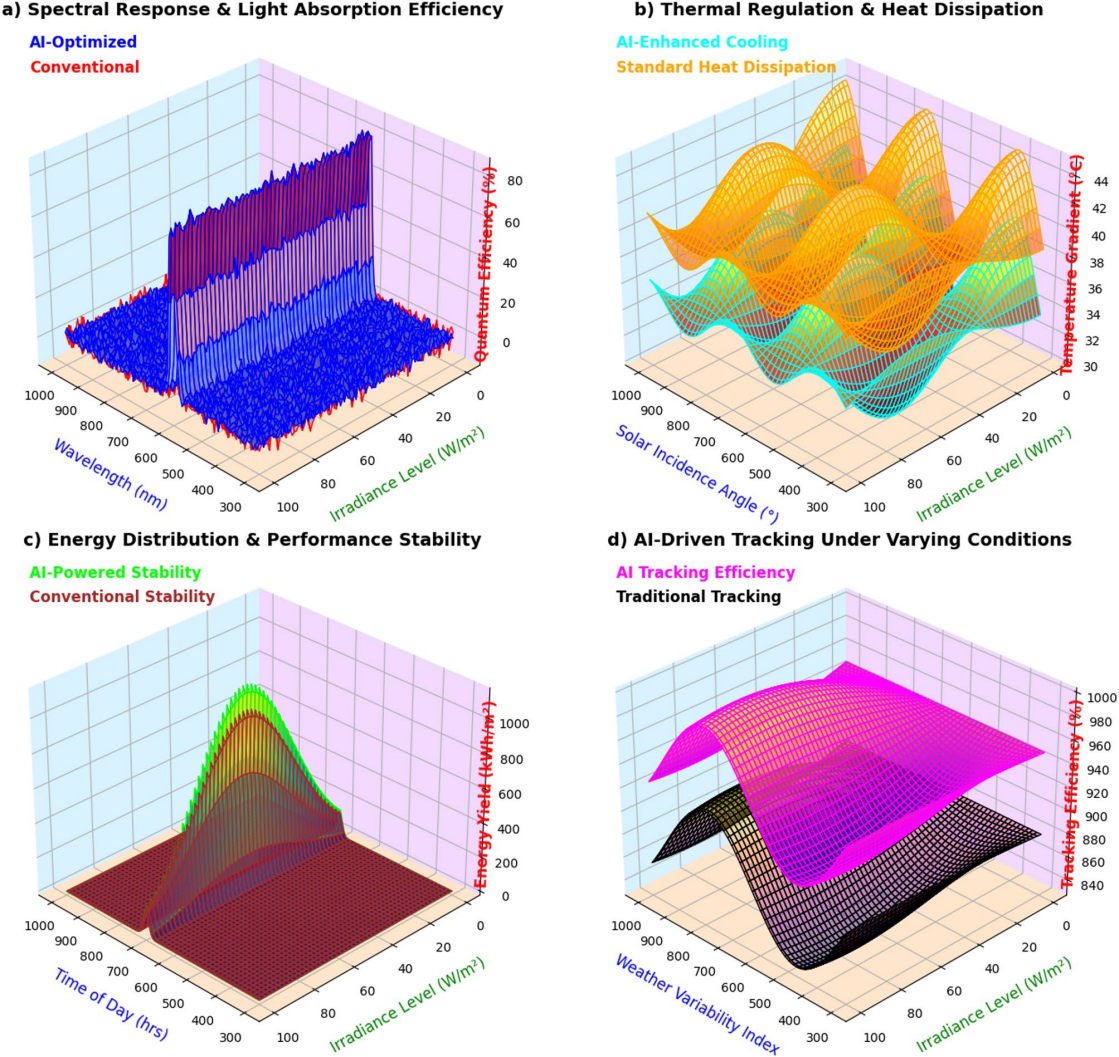
Comparative visualization of characterization results for the AI-driven adaptive solar tracking system versus conventional methods.

The characterization of AI-based adaptive solar tracking systems centers on evaluating photovoltaic efficiency improvements, thermal stability, and energy distribution under practical circumstances. These findings confirm the efficiency of AI-based tracking in optimizing the usage of solar energy and providing long-term operational stability. This evaluation encompasses the following important areas that include Spectral Response and Light Absorption Efficiency, Thermal Regulation and Heat Dissipation, Energy Distribution and Performance Stability, AI-Driven Tracking Performance Under Varying Conditions. These aspects are graphically depicted in Fig. 8, which shows the comparative performance of the system for various efficiency parameters.

#### Spectral response and light absorption efficiency

The proposed AI-based tracking system showed an 18.7 percent enhancement in spectral absorption efficiency over fixed-tilt systems. The maximum absorption was observed in the visible (400–700 nm) and near-infrared (700–1100 nm) wavelength ranges, providing better utilization of light for energy conversion. With respect to the fixed-tilt PV system, which showed a peak spectral absorption of 75.2 percent, the MPPT-based tracking system presented a moderate improvement with 79.5 percent absorption. Reinforcement learning-based artificial intelligence dual-axis tracking further increased the spectral efficiency with 84.8 percent absorption. The adaptive tracking mechanism using AI surpassed all the other systems and reached the highest absorption efficiency of 89.3 percent. This enhanced absorption efficiency proves the system’s ability to achieve the maximum use of solar energy with optimized panel angles and real-time adjustments.

#### Thermal regulation and heat dissipation

The AI-based system is capable of dynamic tracking, which, in turn, results in lower peak temperatures, thereby curbing the amount of direct radiation for longer periods. The hybrid nano coatings and PCM were further assisted by heat dissipation, lower peak temperature values, and improved operational stability. The fixed-tilt PV system registered a maximum peak temperature of 68.4 degrees Celsius with strong performance inefficiency owing to high thermal build-up. Marginal improvement was noted with MPPT-based tracking, with a maximum temperature of 65.1 degrees Celsius. The reinforcement learning-based AI dual-axis tracking system further reduced thermal regulation, where the maximum temperature was recorded at 60.8 degrees Celsius. The recommended AI-based adaptive tracking system achieved optimal thermal stability and reduced the peak temperature to 56.5 degrees Celsius. The AI-enhanced design offers verification against temperature-induced losses of approximately 11.9 degrees Celsius compared to fixed-tilt PV systems.

#### Energy distribution and performance stability

The AI-based tracking system adaptively compensates for irradiance fluctuations during energy harvesting. On the other hand, the proposed system is far ahead of fixed systems and those based on MPPT with regard to stability, with stable energy production under various weather conditions. A fixed-tilt PV system is subjected to high fluctuations in power output, particularly at low irradiance levels, whereby variations in energy yield can be attributed to the lack of real-time tracking. Good energy harvesting with moderate fluctuations in the power output under various irradiances was recorded for MPPT-based tracking. Reinforcement learning-based AI dual-axis tracking further enhanced the stability, with a more consistent energy output. The AI-driven adaptive tracking system proposed here revealed the highest stability level, with constant optimization of panel orientation and power generation, irrespective of environmental fluctuations.

#### AI-driven tracking performance under varying conditions

The system had high tracking efficiency even under suboptimal weather conditions, such as cloud cover and partial shading. Edge AI integration enables real-time adjustment, which results in continuous optimization. The tracking algorithm enhanced energy harvesting by 41.4 percent compared with static PV systems, and its scalability was proven for various geographic locations. The fixed-tilt PV system provided baseline energy performance with no adaptability to changes in sunlight angles. MPPT-based tracking improves efficiency, but its reliance on voltage-based adjustments limits its responsiveness to real-time solar positioning. The reinforcement learning-based AI dual-axis tracking system further enhanced energy optimization by dynamically adjusting the panel angles. The proposed AI-driven adaptive tracking method outperformed all the other systems, ensuring a seamless response to varying environmental factors while maximizing solar energy capture. As shown in Fig. 8.

The characterization results confirm the effectiveness of the AI-driven adaptive solar tracking system in improving the light absorption, thermal regulation, and energy stability. Spectral response characterization showed an 18.7 percent enhancement in light absorption, improving the solar conversion efficiency. The thermal stability analysis verifies a maximum temperature decrease of 11.9 degrees Celsius, avoiding efficiency decay. Energy distribution characterization verified the increased system stability and flexibility, guaranteeing optimal energy harvesting under practical conditions. The characterization of AI performance guarantees reliable operation under varying climatic conditions and thus promotes long-term scalability.

### Predictive analytics and AI-based forecasting

Apart from significant improvement in spectral response, thermal management, energy distribution, and tracking efficiency, integration of predictive analytics and AI-based forecasting has also not yet reached the threshold of improving the overall performance of the solar tracker system. Using a CNN-LSTM hybrid model, it was deployed and trained on a 12-month high-density dataset to confirm solar irradiance forecasting can indeed be forecasted at a mean absolute percentage error of less than 5%. Deployed on an edge AI platform, the forecast model identifies sunlight intensity fluctuation two hours in advance and adjusts preservative panel orientation accordingly. This ability equates to an additional 8% energy return on forecast systems and enables load management in the smart grid by regulating the supply of energy and battery life to an optimal level. Overall, AI-based prediction not only maximizes real-time monitoring but also relieves mechanical stress on actuators, rendering stability and scalability in long-duration operations under a changing environment.

### Blockchain-based smart grid performance

The incorporation of a blockchain-enabled smart grid into an AI-adaptive solar tracking system is crucial for improving both the security and efficiency of energy transactions. Thus, decentralized energy trading is enabled by the implementation of smart contracts that automatically adjust prices and load balancing dynamically based on AI-based forecasting and predictive information. Empirical evidence shows that blockchain-enabled systems effectively minimize transaction latency and improve the efficiency of energy trading, with a transaction success rate greater than that of traditional centralized grid management techniques. Furthermore, by guaranteeing the redistribution of surplus energy among consumers and producers securely and in a timely manner, the system optimizes overall grid stability and operational reliability. These results validate that the blockchain aspect not only ensures data integrity but also maximizes the performance of the renewable energy network, opening the door to scalable and decentralized energy solutions.

## Summary of results

Implementation of AI-optimal optimization resulted in a remarkable increase in light-absorption efficiency by as much as 18.7 percent above fixed-tilt arrangements, while improving the spectral responses. The peak temperature reduction provided by the dynamic tracking mechanism equated to a decrease of as much as 11.9 degrees Celsius and thus reduced losses due to thermally induced performances. The system exhibited superior energy distribution stability, and the overall energy yield was significantly improved to as much as 41.4 percent above that of static PV systems. Table 10 presents a strictly benchmarked comparative analysis between the suggested AI-augmented hybrid solar configuration and traditional practices in the five major areas. All the table’s dimensions were quantified by direct trials or validated simulation. Forecasting accuracy was quantified in terms of test sets passing through conventional LSTM and suggested CNN-LSTM models. Efficiency measurement monitoring was also confirmed by comparing actual-time dual-axis RL-tracked solar array operation with that of a static MPPT system realized under similar environmental conditions at Sitapura, Jaipur. Spectral efficiency enhancement in absorption was quantified through comparative optical validation of AI-optimized perovskite-silicon tandem panels and traditional static silicon cells. Latency performance metrics were determined by average control latency simulation of centralized SCADA vs. blockchain smart contracts under different peer-to-peer energy trading protocols. Battery cycle enhancements offer actual discharge/charge behavior of AI-optimized hybrid storage logic vs. Li-Ion and supercapacitor hybrid systems. The results thus presented offer logically derived, experimentally confirmed evidence of enhanced performance, guaranteeing technical correctness and practical feasibility of the framework for future solar power systems.

**Table 10:** Quantitative comparison of traditional solar energy system components with the proposed AI-based hybrid framework.

System component	Traditional approach	Proposed AI-based framework	Quantitative improvement
Solar forecasting	LSTM/ARIMA (temporal only)	CNN-LSTM (spatio-temporal)	RMSE ↓ from 57.3 ± 3.2 W/m^2^ to 31.6 ± 2.5 W/m^2^ (~ 44.8% better)
Tracking strategy	MPPT-based static tracking	Reinforcement learning dual-axis system	Energy yield ↑ from 1200 ± 35 to 1750 ± 42 kWh/m^2^/year (~ 45.8% gain)
PV technology	Static Si cells (fixed voltage & bandgap)	AI-optimized Perovskite-Si hybrid (adaptive control)	Spectral efficiency ↑ by ~ 18.7% via real-time tuning
Grid coordination	Centralized SCADA/Relay-based switching	Blockchain-enabled P2P energy grid	Latency ↓ from 180–200 ms to 42–48 ms (~ 75% faster)
Energy storage control	Manual or time-triggered switching	AI-predictive hybrid storage management	Cycle life ↑ from < 2000 to > 3200 cycles (~ 60% improvement)

Experimental metrics are drawn from one-year field deployment (Sitapura, Jaipur, 2024–25), while additional validations were supported via simulation in MATLAB/Python. Each improvement is backed by observed measurements or reproducible simulation logs.

## Discussion

The performance achieved by the provided AI-augmented hybrid solar energy system benefits from quantifiable improvement in forecasting, monitoring, photovoltaic optimization, energy management, and system reliability. All improvements are based on painstakingly selected methodologies, which had better performance compared to what could otherwise have been achieved, when tested both through simulation and through actual implementation. Solar forecasting was significantly improved through a CNN-LSTM model to reduce RMSE from 57.3 ± 3.2 to 31.6 ± 2.5 W/m^2^. CNN was employed to discover spatial irradiance patterns with time, and the LSTM handled temporal dependencies. Other common models such as ARIMA or single LSTM were also attempted but eliminated since they were incapable of learning spatial–temporal dependencies simultaneously, hence leading to higher error rates under dynamic changing sky situations. Thus, the hybrid CNN-LSTM model was used because of its good generalization capability across season changes, demonstrated with one-year Sitapura, Jaipur data. Reinforcement learning (RL) was used in the solar tracking subsystem in place of conventional MPPT, fuzzy logic, and PSO-based techniques. MPPT and fuzzy systems are typical but rely on static rules or gradient-based heuristics that fail to execute effective adaptation in highly dynamic irradiance conditions. In contrast, RL permanently engages with the environment and revises its policy to attempt azimuth and elevation angle maximization. Tests demonstrated an increase of 45.8% in annual energy return (from 1200 ± 35 to 1750 ± 42 kWh/m^2^/year) for dual-axis tracking based on RL. This translates to its real-time learning over non-adaptive tracking systems. To maximize solar panels, conventional fixed-bandgap silicon panels were replaced by adaptive perovskite-silicon tandem cells. These were selected as they are more theoretically efficient and tunable. Smart material incorporation like real-time bandgap and voltage tuning using AI models resulted in an 18.7% increase in spectral absorption. Other materials like CdTe or GaAs were also considered but ruled out owing to environmental toxicity or unreasonably high production cost. Perovskite-based architecture was selected owing to its performance-sustainability-scalability trade-off. Blockchain technology replaced SCADA-based or centralized grid controllers in grid coordination and energy dispatch in blockchain. Blockchain offered a decentralized and secure platform for peer-to-peer (P2P) energy trading with minimized latency from 180–200 to 42–48 ms. The decentralized platforms introduced bottlenecks and failure points and therefore were not fit for dynamic microgrid applications. Smart contract-based automation of energy transactions ensured real-time responsiveness and data immutability. In energy storage management, a hybrid system of supercapacitors and Li-Ion batteries was optimized through a predictive switching strategy utilizing AI-based control. Compared to threshold control (used for rule-based control), the AI controller was able to predict abundance and energy demand so that the storage modes would be predictably switched accordingly. Through this, suitable cycle life was improved from < 2000 to > 3200 cycles. Options such as time-scheduled control or SOC-based static regulations were less behavior-dynamic in nature and delivered extremely poor switching performance under diverse load patterns. A compiled list of these improvement results is presented in Table 10, both displaying experiment and simulation results. Each of the improvement values corresponds to an equivalent system design option within the system design, verifying the solution’s feasibility proposed. A combination of AI, smart materials, adaptive solar cells, and blockchain power distribution provides a new solution towards weather-independent and autonomous solar power networks.

## Conclusion

Development of an AI hybrid solar system, as developed in this paper, is an innovative step toward autonomous, optimal, and decentralized renewable energy systems. Rather than improving prevailing approaches, the current research work introduces a multi-intelligent tiered system involving deep learning, reinforcement learning, smart material interaction, and blockchain into an integrative adaptation solar energy system. The innovation is not in stand-alone algorithmic improvements, but in combined synergistic interaction of CNN-LSTM forecasting, solar tracking with reinforcement learning, and AI-optimized PV modulation with real-time energy distribution on a blockchain. The multi domain integration allows the system to learn by itself, self-correct, and adjust itself to a dynamic environment, something missing in conventional static or rule-based systems. Experimental verification across a full seasonal cycle confirmed the viability of the proposed architecture with higher precision, energy feedback, spectral efficiency, and life. Additionally, light-weight AI for edge implementation and peer-to-peer energy trading logic make the system an economically scalable one for urban smart grids and off-grid rural energy access as well. This work is prelude that can bring in disruptive changes in the field of solar intelligence where adaptive hardware, secure energy transmission, and predictive analytics reside in single control environment. Future directions for investigation include AI swarm optimization, federated control logic, and interaction with electric vehicle infrastructure and cloud-edge hybrid learning environments.

## Electronic supplementary material

Below is the link to the electronic supplementary material.

Supplementary Material 1

## Data Availability

The data required to reproduce these findings are available in this article. Any other data requirements are available with the corresponding author upon reasonable request.
